# Stereoretentive Formation of Cyclobutanes from Pyrrolidines: Lessons Learned from DFT Studies of the Reaction Mechanism

**DOI:** 10.1021/acs.joc.3c00080

**Published:** 2023-03-20

**Authors:** Roger Monreal-Corona, Miquel Solà, Anna Pla-Quintana, Albert Poater

**Affiliations:** Institut de Química Computacional i Catàlisi and Departament de Química, Universitat de Girona, C/ Maria Aurèlia Capmany 69, 17003 Girona, Catalonia, Spain

## Abstract

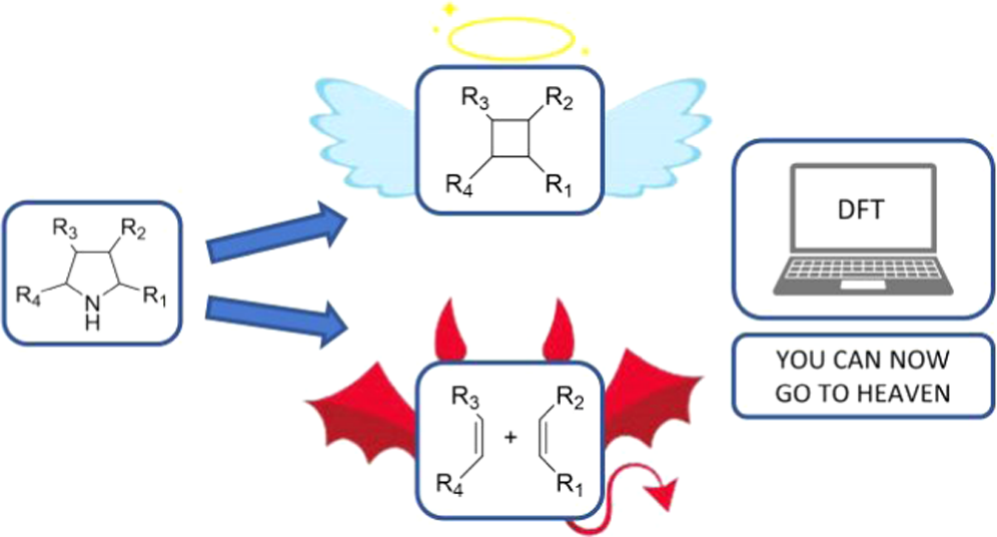

The stereoselective synthesis of cyclobutanes that possess an array of stereocenters in a contiguous fashion has attracted the wide interest of the synthetic community. Cyclobutanes can be generated from the contraction of pyrrolidines through the formation of 1,4-biradical intermediates. Little else is known about the reaction mechanism of this reaction. Here, we unveil the mechanism for this stereospecific synthesis of cyclobutanes by means of density functional theory (DFT) calculations. The rate-determining step of this transformation corresponds to the release of N_2_ from the 1,1-diazene intermediate to form an open-shell singlet 1,4-biradical. The formation of the stereoretentive product is explained by the barrierless collapse of this open-shell singlet 1,4-biradical. The knowledge of the reaction mechanism is used to predict that the methodology could be amenable to the synthesis of [2]-ladderanes and bicyclic cyclobutanes.

## Introduction

Cyclobutane is a four-membered carbocycle that is found at the core of many bioactive and natural products.^1−3^ Many synthetic methods have been reported for the synthesis of multisubstituted cyclobutanes including radical cyclization,^4,5^ Wolff rearrangement of α-diazopentanones,^6−8^ oxidative pinacol rearrangement,^9^ or [2+2] cycloaddition reactions.^10^ However, controlling the stereochemistry in the synthesis of this carbocycle with strained sp^3^ carbons is still very challenging.^11−14^ Therefore, it is an important task for the synthetic community to develop new methodologies for the stereocontrolled preparation of substituted cyclobutanes.

**Scheme 1:**
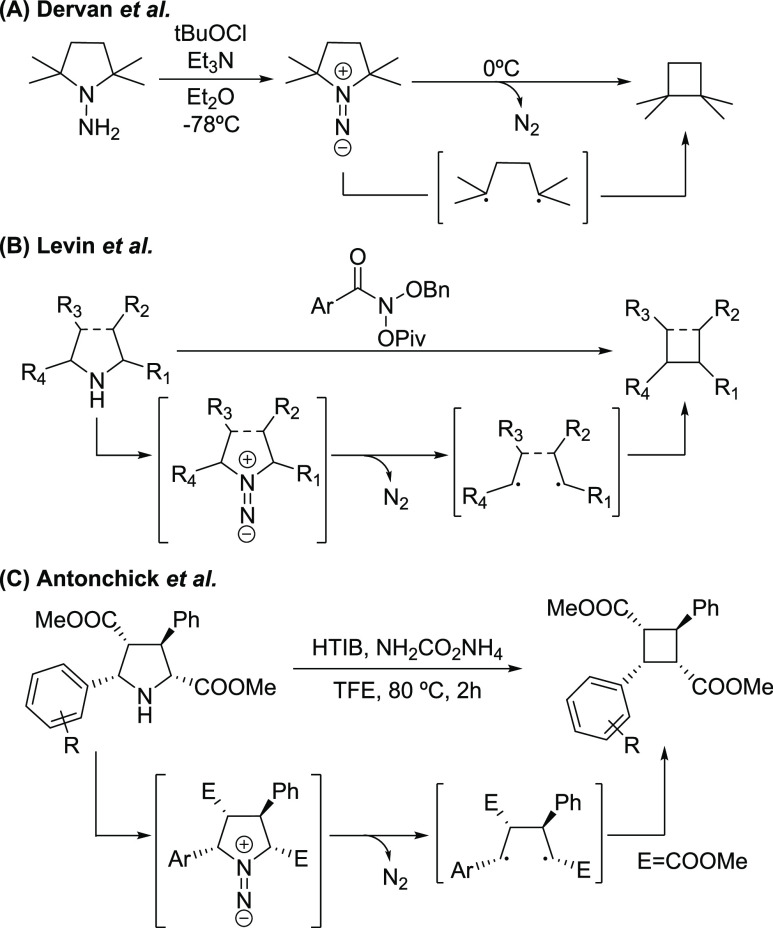
(A) Pioneer Works on the Pyrrolidine Ring Contraction by Nitrogen Extrusion; (B) Nitrogen Deletion of Secondary Amines Using Anomeric Amides; (C) Reaction of Pyrrolidines with an *In Situ* Prepared Iodonitrene Species for the Formation of Cyclobutane Scaffolds

One of the most promising strategies is the synthesis of cyclobutanes by contraction of pyrrolidines (Scheme 1). Dervan and co-workers became pioneers in the field when in 1980 they were able to characterize at −78 °C a 1,1-diazene, formed by oxidation of 1-amino-2,2,5,5-tetramethylpyrrolidine, and identify 1,1,2,2-tetramethylcyclobutane as one of the products formed at 0 °C upon nitrogen extrusion (Scheme 1A).^15^ It was postulated that the thermally generated 1,4-biradical rapidly evolves into the cyclobutane product. 2-Methyl-1-propene was also detected as product, presumably forming upon cleavage of the 1,4-biradical, together with minor amounts of hexenes. Noteworthy, when *C*_2_ symmetrical *trans*-2,5-diethyl-2,5-dimethylpyrrolidylnitrene was reacted, *trans*-cyclobutane was stereospecifically formed.^16^ In 2021, Levin and co-workers showed that *N*-anomeric amides, amides substituted at nitrogen with two electronegative atoms, can act as nitrogen transfer reagent to secondary amines to form 1,1-diazene species, which can then undergo nitrogen extrusion to generate the short-lived diradicals that recombine to form a C–C bond (Scheme 1B). The nitrogen deletion method is applicable to a broad array of aliphatic amines, including cyclic analogues such as pyrrolidines.^17^

In parallel, an emerging research area has shown that a combination of (diacetoxyiodo)benzene (PIDA) and ammonia or its surrogates also promotes the transfer of nitrogen. This combination has been shown to transfer nitrogen to sulfur for the synthesis of NH-sulfoximines^18^ and sulfonimidamides,^19^ and also to transfer nitrogen to nitrogen to access hydrazinium salts^20^ and diazirines.^21^ In these works, it is postulated that iodonitrene, *in situ* generated upon reaction of (diacetoxyiodo)benzene (PIDA) and ammonia, is responsible for the nitrogen transfer. In 2021, Antonchick et al. reported the use of hypervalent iodine(III) reagent and ammonium carbamate to trigger the stereoselective synthesis of cyclobutanes by contraction of pyrrolidines (Scheme 1C).^22^ This new approach to enantiopure cyclobutanes is especially appealing due to the many methods that exist for the asymmetric synthesis of pyrrolidines.^23^

The reaction pathway was hypothesized (Scheme 1C) as an initial nitrene transfer from the *in situ* generated iodonitrene to the pyrrolidine species, followed by the nitrogen extrusion from the 1,1-diazene intermediate to give rise to the 1,4-biradical that can collapse into the desired cyclobutane scaffold.

In this work, we present our endeavors to unveil the reaction mechanism for the stereoretentive synthesis of cyclobutanes by contraction of pyrrolidines and use the obtained mechanistic information to aid the synthesis of fused cyclobutanes.

## Results and Discussion

**Figure 1:**
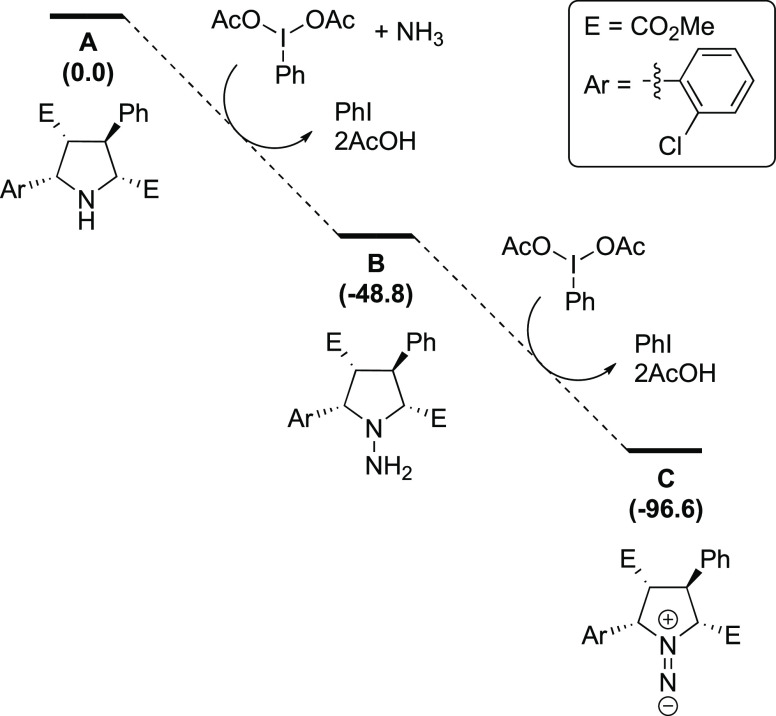
Reaction mechanism for the formation of the 1,1-diazene intermediate **C** calculated at the (U)M06-2X-D3/6-311G(d,p)(smd-2,2,2-trifluoroethanol)//(U)M06-2X-D3/Def2SVP(smd-2,2,2-trifluoroethanol) level of theory. Relative Gibbs energies in kcal/mol.

The preparation of cyclobutane scaffolds from pyrrolidines starts with the transfer of one nitrogen atom to the pyrrolidine species to generate a 1,1-diazene (see Section S1). Antonchick and co-workers postulated,^22^ based on the precedents from the literature,^18−21^ that reaction of hypervalent iodine(III) species with ammonium carbamate *in situ* afforded iodonitrene species, capable of delivering one nitrogen atom to the pyrrolidine. Although the thermodynamics of the transfer of nitrogen from iodonitrene to pyrrolidine was highly exergonic, attempts at studying the thermodynamics toward iodonitrene formation invariably showed highly endergonic processes that rule out its formation. Alternatively, we studied a process encompassing two consecutive oxidations mediated by the hypervalent iodine(III) reagent. In the first one, pyrrolidine and ammonia are oxidized to *N*-aminated pyrrolidine **B** with stabilization energy of 48.8 kcal/mol, and in the second one, the hydrazine moiety in the *N*-aminated pyrrolidine is oxidized to form 1,1-diazene species **C** that is 96.6 kcal/mol more stable than **A** (Figure 1). We assume that in each oxidation reaction, there is an initial ligand exchange followed by the redox step promoted by deprotonation.^24^ This two-step oxidation is in agreement with the methodology employed experimentally that uses 2.5 equivalents of the hypervalent iodine(III) compound. Furthermore, formation of 1,1-diazene from 1,1-disubstituted hydrazines has already been reported,^15,16^ and Antonchick and co-workers^22^ showed that the reaction starting from an *N*-aminated pyrrolidine (analogous to intermediate **B**), under otherwise standard reaction conditions, afforded the corresponding cyclobutane with increased yield compared to the reaction starting from the pyrrolidine.^25^

**Figure 2:**
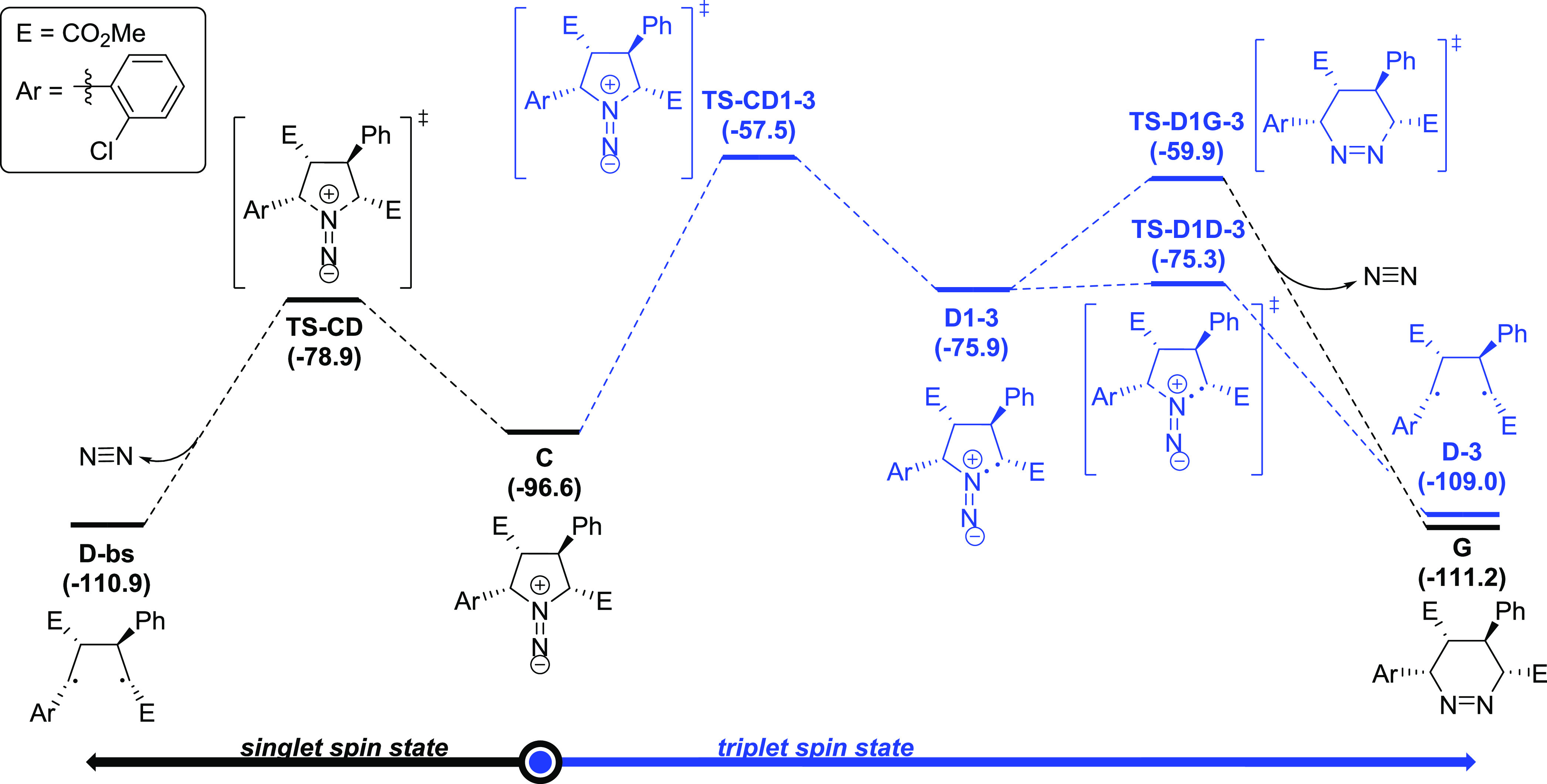
Reaction mechanism for the nitrogen extrusion of 1,1-diazene **C** calculated at the (U)M06-2X-D3/6-311G(d,p)(smd-2,2,2-trifluoroethanol)//(U)M06-2X-D3/Def2SVP(smd-2,2,2-trifluoroethanol) level of theory. Relative Gibbs energies in kcal/mol. Species in black correspond to closed- or open-shell singlet states, whereas species in blue are in their triplet state.

At this point, 1,1-diazene **C** needs to proceed through the extrusion of nitrogen to form the desired cyclobutane scaffold. For the extrusion of nitrogen, we have considered two options, namely, N_2_ extrusion in the singlet (black pathway in Figure 2) and in the triplet (blue pathway in Figure 2) spin states. Focusing on the triplet spin state, the release of nitrogen gas proceeds in a stepwise mechanism in which the first C–N bond is cleaved homolytically to yield species **D1-3** through **TS-CD1-3** with a kinetic cost of 39.1 kcal/mol. The other possibility for the C–N bond cleavage was also considered showing a higher kinetic cost, and it is shown in Figure S3. Then, the second C–N bond is also cleaved through **TS-D1D-3**, which is found to have a kinetic cost of 0.6 kcal/mol to yield the biradical species **D-3** with an energy stabilization of 109.0 kcal/mol compared to starting materials. Moreover, from intermediate species **D1-3**, the reaction may proceed to form tetrahydropyridazine species **G** through **TS-D1G-3** with a Gibbs energy barrier of 16.0 kcal/mol. These reaction paths involving triplet spin states can be discarded due to the high activation barriers compared to the singlet spin state reactivity of **C** (*vide infra*). Indeed, by keeping the multiplicity of the system in the singlet spin state, intermediate **C** can undergo nitrogen extrusion through **TS-CD** by cleaving the two C–N bonds homolytically and simultaneously, with an associated activation energy of 17.7 kcal/mol. Just after the release of N_2_, the system adopts a biradical singlet character that leads to the formation of the 1,4-biradical species **D-bs**, which is 14.3 kcal/mol more stable than the 1,1-diazine intermediate **C**.

When Antonchick and co-workers added TEMPO,^22^ a radical scavenger, to the reaction mixture under the standard conditions, the formation of cyclobutane product was suppressed. Furthermore, the use of 1,1-diphenylethylene or 9,10-dihydroanthracene as radical trapping reagents decreased the yield of cyclobutane product and in the latter case, led to the identification of anthracene in the reaction mixture by gas chromatography–mass spectrometry (GC–MS). These experiments imply a radical nature of the transformation and experimentally support the intermediacy of the 1,4-biradical intermediate **D-bs**. Like in *p*-benzyne,^26^ for the 1,4-biradical intermediate **D-bs**, the existence of a 1,4 interaction explains the higher stability of the open-shell singlet state compared to the triplet state.

**Figure 3:**
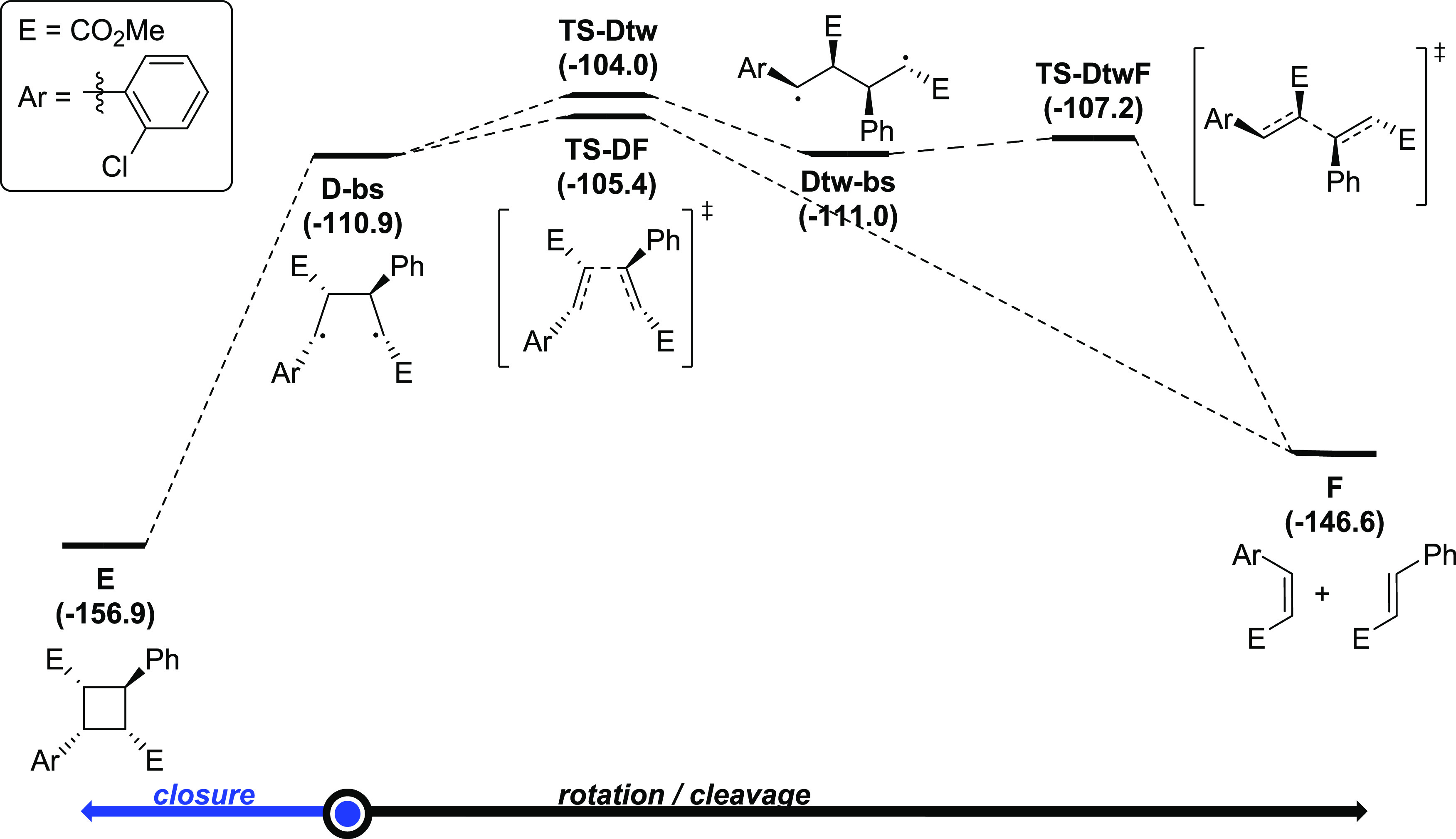
Reaction mechanism for the 1,4-biradical reactivity calculated at the (U)M06-2X-D3/6-311G(d,p)(smd-2,2,2-trifluoroethanol)//(U)M06-2X-D3/Def2SVP(smd-2,22-trifluoroethanol) level of theory. Relative Gibbs energies in kcal/mol.

**Scheme 2:**
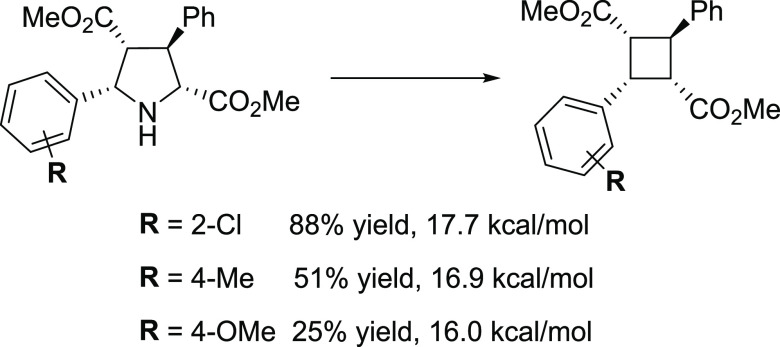
Experimental Yield (%) and Activation Energy of the Rate-Determining Step (rds) (in kcal/mol) for Three Different Derivatives

1,4-Biradical species are known to suffer rotation, cleavage, and/or closure with relative rates that depend on the structure of the substrate and the temperature of the reaction.^27^ Therefore, the three processes were computationally analyzed (Figure 3). The 1,4-biradical intermediate **D-bs**, which has a *gauche* conformation, can yield the desired cyclobutane product **E** (−156.9 kcal/mol) through closure in a process that was found to be barrierless. On the other hand, it can undergo β-fragmentation from the *gauche* conformation to generate alkene byproducts **F** (−146.6 kcal/mol). Alkene byproducts have been experimentally observed in the reaction of pyrrolidines that react sluggishly (*vide infra*, R = 4-OMe in Scheme 2). Species **D-bs** can undergo C–C bond cleavage through **TS-DF** with an energy barrier of 5.5 kcal/mol to yield **F**.

Moreover, **D-bs** can also rotate about the two central carbon atoms of the cyclobutane scaffold to yield **Dtw-bs** in the *trans* conformation, which is 0.1 kcal/mol more stable than **D-bs**, and finally proceed through C–C bond cleavage with a kinetic cost of 3.8 kcal/mol to yield **F**. This case scenario was previously studied computationally by Houk and experimentally by Zewail.^28,29^ These authors reported a similar profile for the tetramethylene biradical, with the only difference being that to go from the biradical species to the cyclobutane product, an energy barrier of about 1 kcal/mol was calculated. In the case under study, closure of the 1,4-biradical in the *gauche* conformation to the cyclobutane is both kinetically and thermodynamically favored.

**Figure 4:**
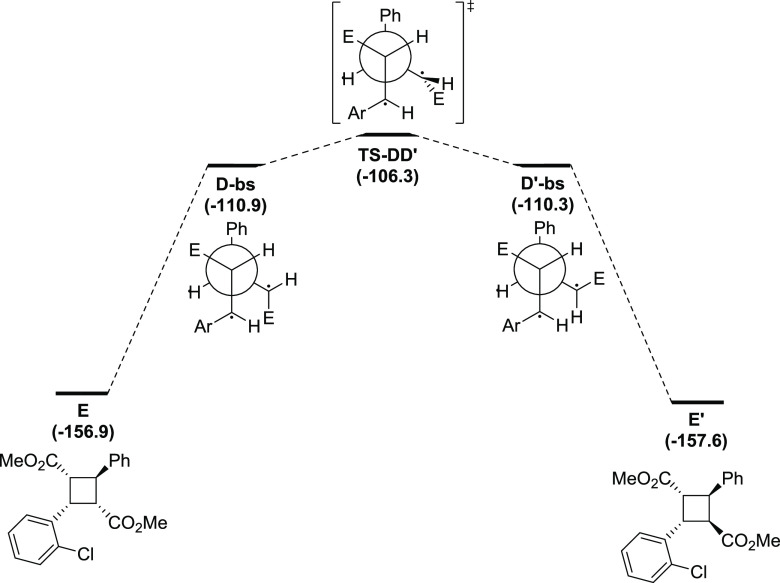
Conformation study in the closure of 1,4-biradicals for the preparation of stereoisomeric cyclobutanes calculated at the (U)M06-2X-D3/6-311G(d,p)(smd-2,2,2-trifluoroethanol)//(U)M06-2X-D3/Def2SVP(smd-2,2,2-trifluoroethanol) level of theory. Relative Gibbs energies in kcal/mol. *E* = CO_2_Me and Ar = PhCl.

A key point on the experimental methodology reported by Antonchick and co-workers^22^ is the stereospecificity of the reaction. A closer look at the geometry of **D-bs** shows that the carbons bearing the radical are *quasi*-planar showing dihedral angles of 173.5 and 178.6°, for the C-Ar and C-E carbons atoms, respectively, which make their hybridization close to sp^2^. For the reaction to give a stereoisomer in which the configuration of the carbon bearing the radical in the intermediate changes, the 1,2- (or 3,4-) bond in the 1,4-biradical moiety needs to rotate. The energy barrier toward this rotation has been computationally evaluated (see Figure 4 for the rotation with the lowest energy barrier and the Supporting Information (SI) for the other possibilities evaluated). The rotation of the PhCH-CHE bond (directing the H towards the inside the 1,4-biradical moiety) has a kinetic cost of 4.6 kcal/mol. Thus, the formation of diastereomer **E’** is unfavorable compared to the barrierless biradical collapse to the stereoretentive product **E** (see Figure 4).^30^

**Figure 5:**
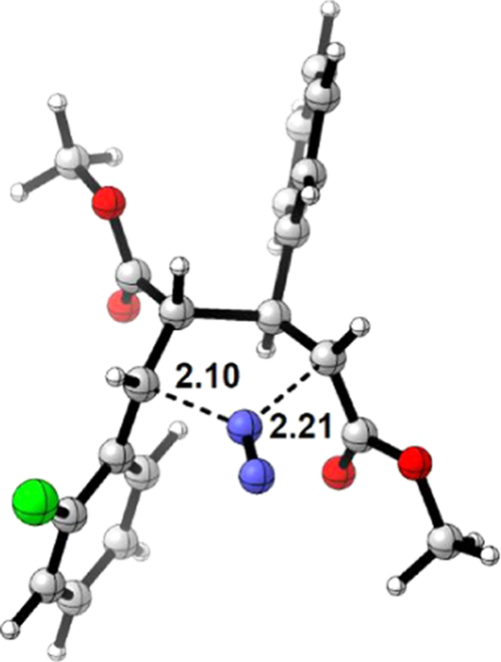
Transition state **TS-CD**; selected distances in Å.

In summary, the mechanism involves an initial highly exergonic formation of 1,1-diazene **C**, followed by the simultaneous cleavage of the two C–N bonds that causes the release of N_2_ to form 1,4-biradical species **D-bs** in the singlet spin state. This process has an activation energy of 17.7 kcal/mol and is the rate-determining step (rds). The optimized structure of **TS-CD** is shown in Figure 5. Finally, a stereoretentive and barrierless ring closure delivers cyclobutane product **E**. The calculated value for the rds agrees with the experimental work showing that although the reaction takes place at 80 °C, it can also proceed at 20 °C, albeit with lower yields of cyclobutane and more competitive oxidation of the substrate, indicating that the reaction barrier can be overcome at 20 °C.

To get more insight into the preparation of cyclobutane scaffolds from pyrrolidines, we computed the activation energy of the rds for three different derivatives that were obtained in significantly different yields experimentally. Results are shown in Scheme 2. For R = 2-Cl, the higher experimental yield is obtained (88%), whereas the calculated barrier for the rds is the higher one (17.7 kcal/mol). On the other hand, for R = 4-OMe, the lower yield is obtained (25% for cyclobutane and a 9% yield for methyl cinnamate, the β-fragmentation product), whereas the kinetic cost is the lowest (16.0 kcal/mol). These results show that no correlation can be obtained between the kinetic cost and the yield. Characterization of the key species **C** was carried out (see Table S1) looking at the electronic effects by means of the highest occupied molecular orbital (HOMO)–lowest unoccupied molecular orbital (LUMO) gap, and at the steric effects by means of %*V*_Bur_ steric indices of Cavallo and co-workers.^31^ No correlation is observed between the mentioned descriptors and the experimental yield, but when we take a look at the total charge of the substituted phenyl ring, a tendency is observed: the more positive the charge of the ring, the lower the experimental yield. Our results show that the low yields obtained experimentally for electron-rich arenes (for example, the phenyl group with R = OMe) are not due to higher activation barriers and, consequently, they must be attributed to the known overoxidation of electron-rich arenes by hypervalent iodine reagents,^32^ and the overall benzylic oxidation.

**Figure 6:**
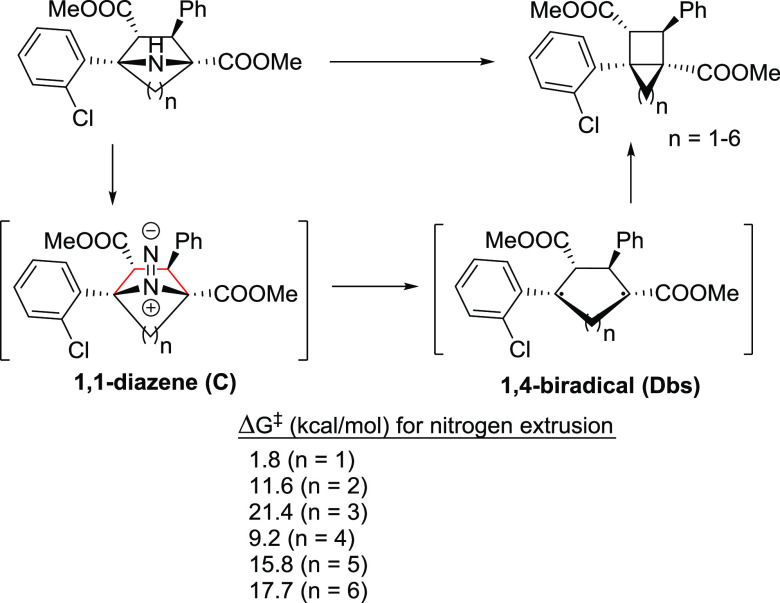
Synthesis of [2]-ladderane (*n* = 2) and bicyclic cyclobutane scaffolds (*n* = 1, 3–6) by contraction of azabicyclo derivatives and activation energy for the rds.

Given the experience of our group in the field of predictive chemistry,^33^ we decided to check if the methodology could be applicable to the synthesis of [2]-ladderanes^3,34^ or other bicyclic structures. To tackle this objective, we envisioned adding an aliphatic chain linking the two carbons in which the biradical is located in species **D-bs**, i.e., studying the reaction in bicyclic 5-azabicyclo[2.1.1]hexane, 7-azabicyclo[2.2.1]heptane, 8-azabicyclo[3.2.1]octane or 9-azabicyclo[4.2.1]nonane derivatives and higher-order analogues (Figure 6).

**Figure 7:**
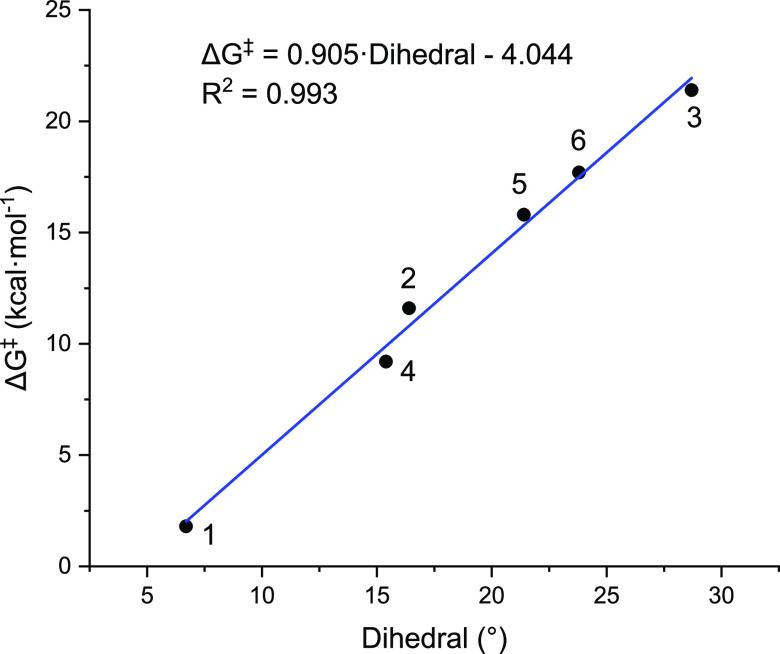
Plot of activation energy (in kcal/mol) vs dihedral angle (°) of species **C** (*n* = 1–6) at the (U)M06-2X-D3/6-311G(d,p)(smd-2,2,2-trifluoroethanol)//(U)M06-2X-D3/Def2SVP(smd-2,22-trifluoroethanol) level of theory.

Based on the structure shown in Figure 5, we computed the activation energy of the rds [i.e., concerted nitrogen extrusion of 1,1-diazene (intermediate of type **C**) to form the 1,4-biradical species (intermediate of type **D-bs**) for the derivatives with *n* = 1–6]. The energy barriers are in the range of 1.8–21.4 kcal/mol, and therefore feasible to be carried out. Of note, we predict that the synthesis of ladderane (*n* = 2) to be easy due to the affordable 11.6 kcal/mol energy barrier for the formation of 1,4-biradical species and a facile ring closure due to easy collapse between the very close in proximity unpaired electrons in the 1,4-biradical intermediate. Of note, 7-azabicyclo[2.2.1]heptane is commercially available, and substituted derivatives thereof can be accessed through procedures described in the literature.^35,36^ In the search for a rationalization of the results by means of a correlation, we plotted the activation energies of the rds against the dihedral angle formed by the two planes that can be defined with the four carbon atoms of the pyrrolidine scaffold in the 1,1-diazene intermediate (atoms in red in Figure 6) to see the impact on the kinetic cost to overcome **TS-CD** (Figure 7). A clear trend is observed in which the lower the dihedral angle, the lower the activation energy to extrude N_2_ and form the 1,4-biradical intermediate. A linear model can be found for Gibbs energy (in kcal/mol): assigning a base value for Δ*G*^‡^ of −4.04 kcal/mol and adding the term 0.905 × dihedral (in °), displaying a nearly perfect *r*^2^ value of 0.993. This good agreement, within the framework of predictive chemistry, expresses how density functional theory (DFT) calculations can contribute to the stereo- or regio-selective achievement of cyclobutanes as recently demonstrated by Houk et al.^37^

## Conclusions

In this work, we have disclosed the mechanism for the stereoselective synthesis of cyclobutanes by contraction of pyrrolidines by means of DFT calculations. The rds of the studied transformation is the simultaneous cleavage of the two C–N bonds that causes the release of N_2_. The stereoretentive pathway of the reaction has been rationalized based on the higher energy required for the rotation of the radicals compared to the cyclization. Activation barriers do not explain the low yields obtained by pyrrolidines containing electron-rich arenes. This result reinforces the conclusion that the low yields are due to the known overoxidation of electron-rich arenes by hypervalent iodine reagents. In a predictive chemistry effort, we have shown that by adding an aliphatic chain linking the two carbons in which the 1,4-biradical is located, the synthesis of [2]-ladderanes and cyclobutane bicyclic analogues is kinetically feasible. Furthermore, the newly revealed mechanistic underpinnings showing a strong correlation of the dihedral angle in the 1,1-diazene with the nitrogen extrusion activation energy will provide guidance for the future rational design of new cyclobutane derivatives.

## Computational Details

All theoretical calculations were performed in the frame of density functional theory (DFT) by means of the Gaussian16 software package.^38^ For geometry optimizations, the hybrid M06-2X Minnesota functional^39^ was used together with the Grimme D3 correction term for the electronic energy.^40,41^ For the basis set, the split-valence basis set with the polarization of Ahlrichs and co-workers (Def2SVP)^42^ was adopted. The method of calculation was chosen on the basis of a benchmark of different functionals (see Table S3). Open-shell singlet states were treated with the unrestricted methodology. The nature of the located structures was confirmed by frequency calculations.^43^ In addition, the intrinsic reaction coordinate (IRC) procedure was used to confirm the two minima connected by each transition state.^44^ Implicit solvent effects were included to simulate 2,2,2-trifluoroethanol (TFE) by means of the solvation model based on density (SMD) continuum solvation model in which the quantum mechanical charge density of the solute interacts with the solvent represented by a polarizable continuum with dielectric constant ε.^45^ Single point energy calculations with the M06-2X-D3 functional and the 6-311G(d,p) basis set^46^ were performed to improve accuracy, again taking into account explicit solvent effects using the SMD model. The reported Gibbs energies in this work include electronic energies obtained at the (U)M06-2X-D3/6-311G(d,p)(smd-TFE)//(U)M06-2X-D3/Def2SVP(smd-TFE) level of theory corrected, with zero-point energies, thermal corrections, and entropy effects evaluated at 353.15 K with the (U)M06-2X-D3/Def2SVP(smd-TFE) method.

## Data Availability

The data underlying this study are available in the published article and its Supporting Information.

## Supporting Information Available

Discussion on alternative mechanisms, properties of intermediate **C**, and *xyz* coordinates and absolute energies of all computed species (PDF)
