# Supplementary material for: Stereoretentive Formation of Cyclobutanes from Pyrrolidines: Lessons Learned from DFT Studies of the Reaction Mechanism
Source: J Org Chem. 2023 Mar 20;88(7):4619–26. doi: 10.1021/acs.joc.3c00080 (PMC10088030; doi:10.1021/acs.joc.3c00080)
Supplement: Supplementary file 1 — jo3c00080_si_001.pdf [file jo3c00080_si_001.pdf]

Stereoretentive Formation of Cyclobutanes from Pyrrolidines: Lessons learned from DFT studies of the reaction mechanism

Roger Monreal-Corona, Miquel Solà,* Anna Pla-Quintana,* and Albert Poater*

Institut de Química Computacional i Catàlisi and Departament de Química, Universitat de Girona, C/ Maria Aurèlia Capmany, 69, 17003 Girona, Catalonia, Spain

Electronic Supplementary Information

(47 pages)

Contents:

Section S1. Mechanism Discussion

Figure S1. Gibbs energy profile (in kcal/mol) for the formation of the active iodonitrene species **III** from PIDA.

Figure S2. Gibbs energy profile (in kcal/mol) for the formation of 1,1-diazene species **C** from the reaction of pyrrolidine species **A** and active species **III**.

Figure S3. Gibbs energy profile (in kcal/mol) of the two possible paths in the triplet spin state for the cleavage of the C-N bond from intermediate **C**.

Figure S4. Gibbs energy profile (in kcal/mol) of the possible rotations to yield the different stereoisomers of product **E**.

Table S1. Summary of the activation energy (in kcal/mol), HOMO and LUMO energies (in a.u.) and chemical hardness of species **C** of the different derivatives at the M06-2X-D3/6-311G(d,p)(SMD-2,2,2-trifluoroethanol) level of theory.

Table S2. Summary of the activation energy (in kcal/mol) and dihedral angle (°) of species **C** of the different ladderane derivatives at the (U)M06-2X-D3/6-311G(d,p)(smd-2,2,2-trifluoroethanol)//(U)M06-2X-D3/Def2SVP)(smd-2,22-trifluoroethanol) level of theory.

Table S3. Benchmark study (in kcal/mol) of the relative electronic energy between species **E** and **F** at the Functional/6-311G(d,p)(smd-2,2,2-trifluoroethanol) level of theory.

Table S4. M06-2X-D3/Def2SVP-SMD(2,2,2-TriFluoroEthanol) Cartesian coordinates of the optimized geometries of the species calculated in this study.

Section S1. Mechanism Discussion

Ammonium carbamate is known to be in equilibrium with the formation of carbon dioxide and two equivalents of ammonia. Upon addition of (diacetoxyiodo)benzene (PIDA) (species **I**) a ligand exchange can take place to form intermediate **II**. The released acetate can deprotonate the quaternary ammonium cationic species to form the active iodonitrene species **III** as shown in Figure S1.

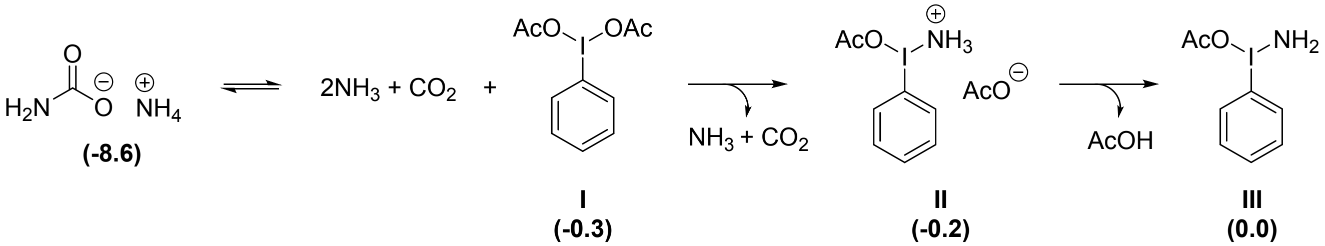

Figure S1. Gibbs energy profile (in kcal/mol) for the formation of the active iodonitrene species **III** from PIDA.

The path for the formation of hypervalent iodine species **III** is endergonic by 8.6 kcal/mol, indicating that this process is possible at room temperature. Experimental evidence of species **III** was reported by Luisi and Bull,[18a] for which they were able to detect the reported species by means of flow high resolution mass spectrometry (HRMS) and ^{15}N labelling.

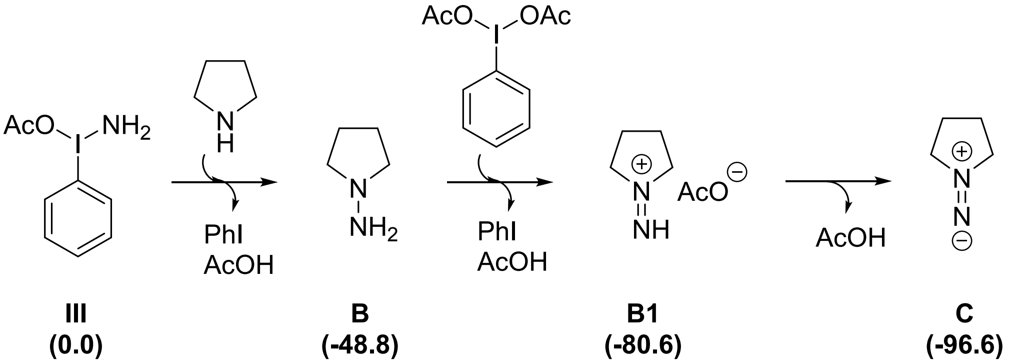

Figure S2. Gibbs energy profile (in kcal/mol) for the formation of 1,1-diazene species **C** from the reaction of pyrrolidine species **A** and active species **III**.

Formation of 1,1-diazene (intermediate **C**) is exergonic by 96.6 kcal/mol starting from intermediate **III** (see Figure S2). As shown in Figure 2 of the manuscript, once C is formed, loss of N2 leads to the 1,4-biradical intermediate in the singlet ground state. The reaction mechanism in the triplet excited state is depicted in Figure S3.

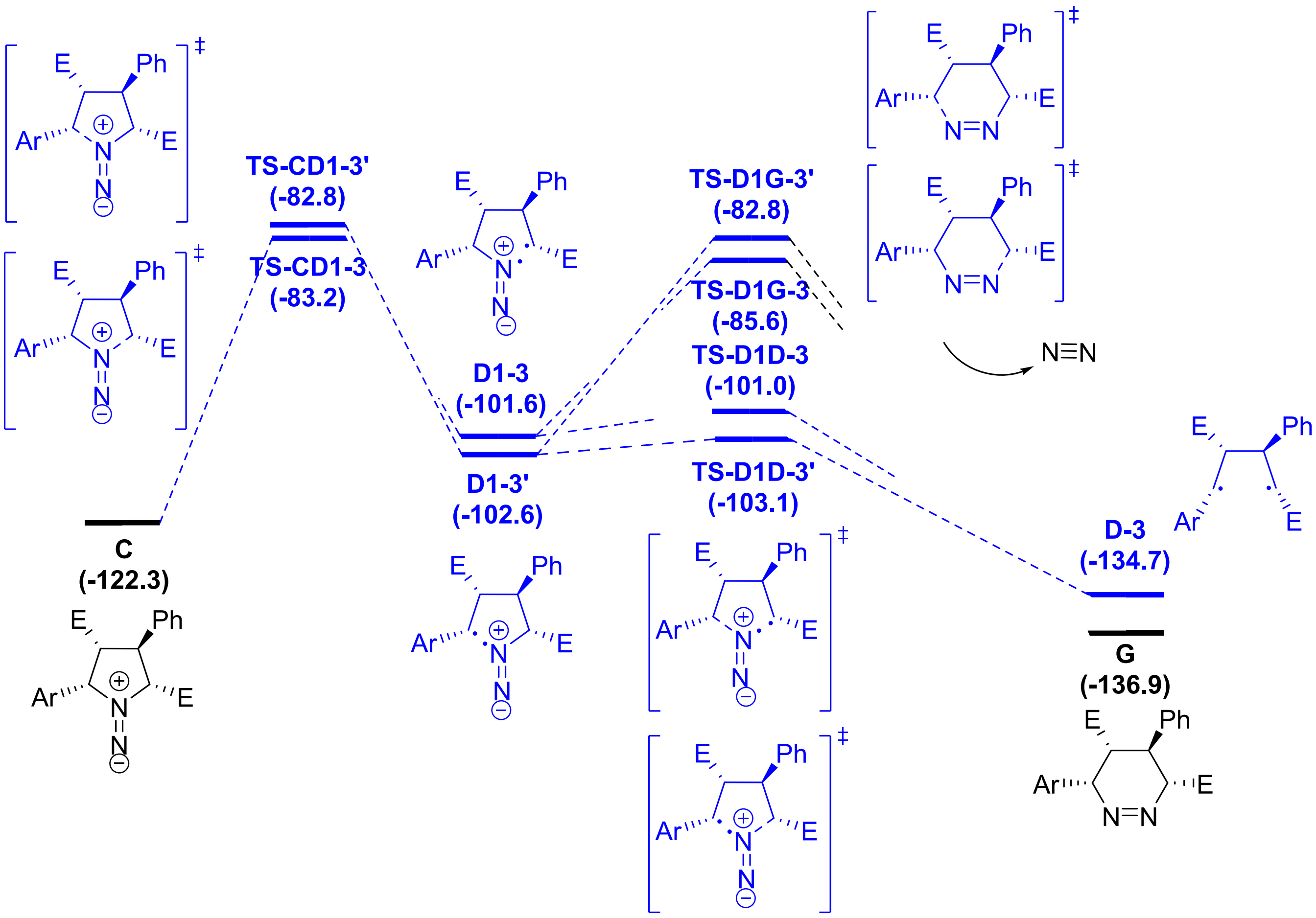

Figure S3. Gibbs energy profile (in kcal/mol) of the two possible paths in the triplet spin state for the cleavage of the C-N bond from intermediate **C.**

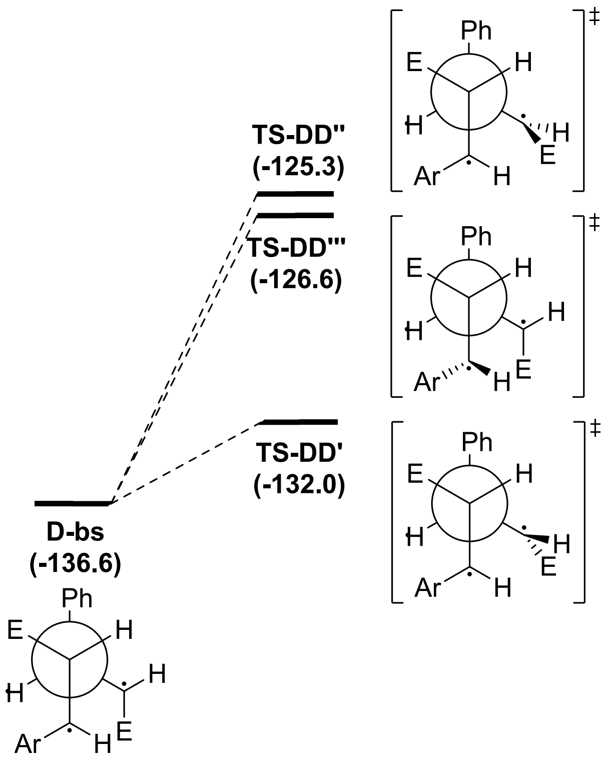

Figure S4. Gibbs energy profile (in kcal/mol) of the possible rotations to yield the different stereoisomers of product **E**.

Given the bulkiness of the Ar substituent and the π-π stacking interaction between the two phenyl units, the rotation about the ArCH-CHE bond is constrained and the phenyl ring is not able to spin. Because of geometry constrains, only one rotation about this mentioned bond, namely ArCH-CHE, was obtained being **TS-DD'''**.

Table S1. Summary of experimental yield (in %), activation energy (in kcal/mol), HOMO and LUMO energies (in a.u.), chemical hardness (in a.u.), $\%V_{bur}$ (in %) and total ring charge (Natural Population Analysis in electrons) of species **C** of the different derivatives at the M06-2X-D3/6-311G(d,p)(SMD-2,2,2-TriFluoroEthanol) level of theory.

Species C	Yield (%)	$\Delta G^‡$ (kcal/mol)	HOMO (a.u.)	LUMO (a.u.)	Chemical Hardness	$\%V_{bur}$ (%)	Ring charge
**2-Cl**	88	17.7	-0.26567	-0.00268	0.263	76.9	-0.01045
**4-Me**	51	16.9	-0.26266	0.00145	0.264	73.3	0.00050
**4-OMe**	25	16.0	-0.26300	0.00137	0.264	73.3	0.00374

Table S2. Summary of the activation energy (in kcal/mol) and dihedral angle (°) of intermediate species **C** towards the synthesis of [2]-ladderane and bicyclic cyclobutene derivatives at the (U)M06-2X-D3/6-311G(d,p)(smd-2,2,2-trifluoroethanol)//(U)M06-2X-D3/Def2SVP(smd-2,22-trifluoroethanol) level of theory.

n	$\Delta G^‡$ (kcal/mol)	Dihedral (°)
**1**	1.8	6.7
**2**	11.6	16.4
**3**	21.4	28.7
**4**	9.2	15.4
**5**	15.8	21.4
**6**	17.7	23.8

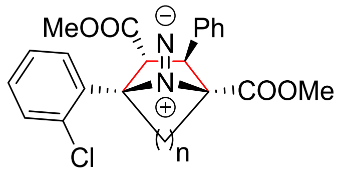

Table S3. Benchmark study (in kcal/mol) of the relative electronic energy between species **E** and **F** at the Functional/6-311G(d,p)(smd-2,2,2-trifluoroethanol) level of theory.

Functional	E (E)	E (F)
**CCSD(T)**	0.0	7.4
**M06-2X-D3**	0.0	10.3
**B3LYP-D3**	0.0	-1.4
**CAM-B3LYP-D3**	0.0	-4.3

Table S4. M06-2X-D3/Def2SVP-SMD(2,2,2-TriFluoroEthanol) Cartesian coordinates and absolute energies (in a.u.) of the optimized geometries of the species calculated in this study.

26			
I SCF Done: -699.812868518 A.U.			
I	0.000013	-1.187086	-0.000007
C	-0.000027	0.953070	0.000026
C	-0.758508	1.611564	-0.956916
C	0.758438	1.611588	0.956972
C	-0.752429	3.007701	-0.946559
H	-1.343316	1.058669	-1.693498
C	0.752339	3.007717	0.946602
H	1.343271	1.058692	1.693534
C	-0.000053	3.702753	0.000016
H	-1.337802	3.548226	-1.692129
H	1.337710	3.548263	1.692158
H	-0.000055	4.793885	0.000009
C	2.919557	-0.543905	-0.286143
C	4.328979	-0.520737	0.242113
H	4.376682	0.191021	1.079387
H	4.601085	-1.511633	0.628484
H	5.020336	-0.210030	-0.548642
O	2.602791	-0.112871	-1.378680
O	2.059389	-1.050805	0.570662
C	-2.919532	-0.544003	0.286138
C	-4.328917	-0.520658	-0.242218
H	-4.601073	-1.511477	-0.628750
H	-5.020315	-0.209982	0.548512
H	-4.376480	0.191202	-1.079417
O	-2.059352	-1.050869	-0.570682
O	-2.602804	-0.113180	1.378767
4			
NH3 SCF Done: -56.5468568594 A.U.			
N	0.000000	0.000000	0.125229
H	-0.000000	0.932434	-0.292201
H	-0.807511	-0.466217	-0.292201
H	0.807511	-0.466217	-0.292201
30			
II SCF Done: -756.375785210 A.U.			
C	3.792244	-1.643789	-0.935331
C	4.511204	-1.347259	0.222366
C	3.900980	-0.662805	1.274079
C	2.564126	-0.275890	1.177798
C	1.865718	-0.581795	0.013381
C	2.456391	-1.255311	-1.051229
H	4.269911	-2.173799	-1.760868
H	5.556332	-1.650155	0.305266
H	4.462934	-0.429028	2.179787
H	2.078967	0.260480	1.995298
H	1.892849	-1.475727	-1.960148
I	-0.168087	0.013541	-0.135361
O	0.793958	2.003638	-0.057658
C	-0.096756	2.948517	-0.062522
O	-1.299923	2.716303	-0.115987
C	0.467395	4.344185	-0.007791

H	1.066592	4.521714	-0.912098
H	-0.343208	5.078131	0.054545
H	1.135781	4.435346	0.858990
H	-1.664936	-2.277925	0.132268
H	-0.435020	-2.596070	-1.039290
H	-0.035310	-2.648975	0.572074
N	-0.615163	-2.169384	-0.124829
O	-2.921867	-0.208070	-0.369598
C	-3.622263	-1.114682	0.136895
O	-3.179110	-2.241414	0.495874
C	-5.097208	-0.831155	0.356724
H	-5.649179	-1.747591	0.596070
H	-5.194566	-0.122436	1.193148
H	-5.521871	-0.349700	-0.534402

8
AcOH SCF Done: -229.065312145 A.U.

C	-1.387422	-0.123386	-0.000024
H	-1.920027	0.832555	-0.001417
H	-1.658218	-0.714300	-0.886070
H	-1.658253	-0.711553	0.887861
C	0.089024	0.116965	-0.000216
O	0.788190	-1.020861	0.000175
H	1.738153	-0.806235	0.000419
O	0.622902	1.200618	-0.000094

22
III SCF Done: -527.291512165 A.U.

C	1.290396	0.183534	0.002790
C	2.355825	-0.208119	-0.798573
C	1.334337	1.317619	0.805627
C	3.511055	0.576842	-0.795922
H	2.300019	-1.102524	-1.422792
C	2.496081	2.090402	0.794046
H	0.477459	1.602561	1.416479
C	3.580652	1.721441	-0.002780
H	4.355039	0.287954	-1.424445
H	2.549252	2.984186	1.417936
H	4.485694	2.331097	-0.005978
I	-0.456961	-1.050836	0.052452
H	1.199956	-2.966851	-0.489194
H	1.222865	-2.691169	1.139147
N	0.607734	-2.799236	0.328163
O	-1.384226	1.154031	-0.288462
C	-2.656434	1.150484	-0.165976
O	-3.327091	0.134510	0.058000
C	-3.329116	2.502442	-0.309724
H	-2.929777	3.188275	0.451120
H	-3.086165	2.927087	-1.294122
H	-4.415804	2.412162	-0.198459

46
A SCF Done: -1589.90506059 A.U.

C	1.150596	1.531267	-0.809916
C	-0.110080	-0.518641	-0.794898
C	0.418967	-0.244344	0.652271
C	1.610221	0.704614	0.408520
H	2.039033	1.848388	-1.384933

H	0.417075	-1.417324	-1.151339
H	0.712143	-1.181943	1.140367
H	1.766962	1.360715	1.274807
N	0.305030	0.639786	-1.579741
H	-0.445553	1.117062	-2.066972
C	2.876475	-0.090186	0.161251
C	3.706935	-0.402869	1.244905
C	3.221791	-0.563647	-1.110710
C	4.856758	-1.170231	1.064404
H	3.448273	-0.034173	2.240797
C	4.373316	-1.331577	-1.292527
H	2.582943	-0.331546	-1.966436
C	5.193509	-1.637943	-0.206901
H	5.495208	-1.399619	1.919771
H	4.629564	-1.691449	-2.290857
H	6.094672	-2.236797	-0.350965
C	-1.596089	-0.812388	-0.825204
C	-2.083050	-2.094538	-0.534477
C	-2.542546	0.200237	-1.022715
C	-3.445455	-2.373205	-0.470380
C	-3.911552	-0.060680	-0.964453
H	-2.203580	1.222081	-1.204381
H	-3.776769	-3.386911	-0.242874
H	-4.622519	0.750837	-1.126279
C	-0.667927	0.400372	1.472045
C	0.513196	2.846027	-0.381193
O	-0.651897	3.082501	-0.968248
O	-1.390061	-0.493596	2.133208
O	1.051102	3.628005	0.365516
O	-0.910577	1.586397	1.486999
C	-1.327767	4.275477	-0.576535
H	-2.258343	4.303197	-1.151869
H	-1.544063	4.242606	0.500166
H	-0.712305	5.156225	-0.802156
C	-2.558373	-0.008873	2.794071
H	-3.227418	0.476336	2.069312
H	-3.044032	-0.884837	3.234971
H	-2.283599	0.710336	3.576992
Cl	-0.967234	-3.397784	-0.197378
C	-4.364771	-1.349138	-0.691221
H	-5.433384	-1.564205	-0.642330

48
B SCF Done: -1643.88081582 A.U.

C	-1.403606	1.046355	0.418292
C	0.775918	0.334097	0.712296
C	0.099106	-0.768477	-0.158463
C	-1.334627	-0.244419	-0.404137
H	-1.756302	0.843299	1.452204
H	0.646840	0.037122	1.771752
H	0.121730	-1.725730	0.374501
H	-1.413303	0.043967	-1.459753
C	-2.448084	-1.212313	-0.081025
C	-3.487324	-1.420817	-0.993477
C	-2.479943	-1.890203	1.145262
C	-4.535420	-2.292395	-0.691502
H	-3.475340	-0.889364	-1.947854
C	-3.524958	-2.760918	1.448845

H	-1.677806	-1.733963	1.872470
C	-4.556439	-2.965280	0.529421
H	-5.337954	-2.445705	-1.415557
H	-3.534703	-3.283121	2.407460
H	-5.374608	-3.647971	0.766246
C	2.249272	0.530541	0.442818
C	3.202808	-0.355757	0.956486
C	2.702163	1.549920	-0.401218
C	4.558896	-0.236054	0.662759
C	4.055695	1.688127	-0.704536
H	1.966295	2.238025	-0.818556
C	4.985372	0.795884	-0.171355
H	5.267321	-0.949864	1.084513
H	4.383962	2.494957	-1.361616
C	0.874796	-0.923384	-1.445937
C	-2.346077	2.065533	-0.195122
O	-2.409656	3.179982	0.521539
O	1.810156	-1.858995	-1.344096
O	-2.977813	1.897447	-1.209588
O	0.729470	-0.240914	-2.432605
C	-3.265029	4.210345	0.023707
H	-3.187224	5.039288	0.733572
H	-2.935270	4.528133	-0.974157
H	-4.300051	3.848107	-0.030149
C	2.744291	-1.945398	-2.418089
H	3.265235	-0.984856	-2.541897
H	3.454297	-2.731434	-2.142606
H	2.230816	-2.205361	-3.353347
H	6.046774	0.896219	-0.403979
Cl	2.691202	-1.681687	1.975268
N	-0.019835	1.526894	0.442964
N	0.271579	2.530842	1.383944
H	-0.085059	3.416066	1.028533
H	-0.236396	2.344235	2.258156
12			
PhI SCF Done: -242.968671482 A.U.			
C	0.000000	1.214415	-1.256738
C	0.000000	-0.000000	-0.572212
C	-0.000000	-1.214415	-1.256738
C	0.000000	-1.206763	-2.652507
C	0.000000	-0.000000	-3.352211
C	0.000000	1.206763	-2.652507
H	0.000000	2.160031	-0.713116
H	-0.000000	-2.160031	-0.713116
H	0.000000	-2.155329	-3.191501
H	0.000000	-0.000000	-4.443041
H	0.000000	2.155329	-3.191501
I	-0.000000	0.000000	1.560561
66			
B1 SCF Done: -2116.02235033 A.U.			
C	2.642569	1.006022	-0.904132
C	1.129674	-0.935782	-1.148704
C	2.505634	-1.355698	-0.576366
C	3.090247	-0.087559	0.054896
H	3.267348	1.036148	-1.813059
H	0.956651	-1.363957	-2.142221

H	3.136383	-1.706874	-1.405982
H	2.595987	0.084363	1.021935
C	4.582834	-0.126180	0.290932
C	5.078606	0.029296	1.588684
C	5.480632	-0.322321	-0.765399
C	6.451941	-0.011721	1.829692
H	4.379388	0.182282	2.413868
C	6.853264	-0.361308	-0.525141
H	5.108573	-0.446485	-1.786408
C	7.341729	-0.206551	0.773403
H	6.827123	0.109650	2.847414
H	7.544287	-0.514017	-1.356038
H	8.416527	-0.238047	0.961063
C	-0.045951	-1.211615	-0.238461
C	-0.907515	-2.288270	-0.485285
C	-0.253305	-0.454683	0.923080
C	-1.937711	-2.611990	0.396679
C	-1.277990	-0.770666	1.810130
H	0.371890	0.419117	1.120425
C	-2.116055	-1.854279	1.550149
H	-2.594440	-3.452387	0.168884
H	-1.424321	-0.167308	2.706980
C	2.364760	-2.483442	0.421622
C	2.623300	2.404340	-0.297636
O	2.316877	3.307466	-1.208596
O	1.914733	-3.578974	-0.165711
O	2.937089	2.629943	0.838685
O	2.592855	-2.389159	1.601296
C	2.212388	4.660707	-0.751878
H	1.930005	5.252931	-1.626817
H	1.440633	4.722713	0.025790
H	3.180545	4.992694	-0.356135
C	1.575247	-4.673214	0.686398
H	0.824042	-4.353966	1.422508
H	1.166596	-5.450862	0.034104
H	2.468996	-5.040413	1.207839
H	-2.927961	-2.101454	2.237531
Cl	-0.737335	-3.260199	-1.922913
N	1.301153	0.539038	-1.374064
N	0.418296	1.188691	-1.896939
H	0.684576	2.189789	-1.953178
I	-3.303713	0.667574	-1.494933
C	-4.412416	0.167411	0.254968
C	-5.247437	-0.947889	0.236421
C	-4.246615	0.935802	1.405851
C	-5.926482	-1.304095	1.403667
H	-5.364533	-1.541207	-0.672113
C	-4.932342	0.565134	2.564933
H	-3.580974	1.802819	1.405165
C	-5.768126	-0.552477	2.568497
H	-6.580924	-2.177911	1.396823
H	-4.805865	1.159367	3.472150
H	-6.298690	-0.836391	3.479011
C	-0.845215	2.992661	0.960379
C	-0.356952	2.958807	2.406439
H	-0.733375	2.038103	2.880373
H	0.739670	2.957395	2.462935
H	-0.767559	3.808785	2.968164

O	-2.021461	3.356753	0.750643
O	-0.029783	2.626514	0.070444

46
C SCF Done: -1643.97154105 A.U.

C	1.411765	1.134677	-0.753659
C	-0.972288	0.492268	-1.010437
C	-0.086470	-0.725983	-0.659535
C	1.166005	-0.148289	0.022319
H	1.894843	0.961592	-1.724694
H	-1.363169	0.419565	-2.030221
H	0.187091	-1.245306	-1.589465
H	0.893135	0.130424	1.051863
N	0.010245	1.666969	-1.026027
C	2.331851	-1.106935	0.077370
C	2.796675	-1.568379	1.313225
C	2.949441	-1.563819	-1.094460
C	3.862680	-2.466747	1.379748
H	2.312711	-1.222482	2.229524
C	4.014378	-2.460431	-1.028840
H	2.594498	-1.218884	-2.069436
C	4.474403	-2.913966	0.209260
H	4.214885	-2.818397	2.351261
H	4.487436	-2.807549	-1.949299
H	5.308574	-3.616206	0.259695
C	-2.089657	0.761562	-0.033008
C	-3.402657	0.341154	-0.284375
C	-1.828604	1.370566	1.202903
C	-4.419098	0.512266	0.655683
C	-2.832844	1.547504	2.149787
H	-0.817826	1.724485	1.419897
H	-5.428859	0.174203	0.420724
H	-2.599837	2.025775	3.101947
C	-0.809026	-1.708264	0.232898
C	2.144372	2.195110	0.033243
O	3.167176	2.702382	-0.628202
O	-1.752927	-2.355552	-0.432035
O	1.818267	2.535934	1.143972
O	-0.593725	-1.860559	1.410826
C	3.917538	3.717470	0.043036
H	4.716828	4.008355	-0.644602
H	3.270847	4.575878	0.266861
H	4.338234	3.319108	0.975516
C	-2.622879	-3.188366	0.332362
H	-3.116675	-2.596604	1.116331
H	-3.362179	-3.584015	-0.370758
H	-2.057431	-4.008404	0.794676
N	-0.281710	2.813193	-1.194211
C	-4.130165	1.114110	1.876871
H	-4.924871	1.247576	2.612464
Cl	-3.824166	-0.412996	-1.801380

46
TS-CD1-3 SCF Done: -1643.90265718 A.U.

C	1.070796	-1.388969	0.405532
C	-1.252534	-0.314083	0.787288
C	-0.114238	0.693623	0.646543
C	1.060982	0.056177	-0.107100

H	1.268191	-1.414128	1.484649
H	-1.424824	-0.644599	1.810552
H	0.215702	0.938382	1.668648
H	0.814564	0.009187	-1.180206
N	-0.306631	-1.930994	0.201505
C	2.391919	0.750739	0.056404
C	3.103883	1.164215	-1.073998
C	2.957823	0.939664	1.323795
C	4.358385	1.761526	-0.942899
H	2.665612	1.019293	-2.064321
C	4.210254	1.536788	1.455998
H	2.418941	0.613040	2.217333
C	4.913995	1.948919	0.322447
H	4.902063	2.081774	-1.833592
H	4.640591	1.678451	2.449072
H	5.895066	2.415821	0.427095
C	-2.451475	-0.243298	-0.047476
C	-3.709809	-0.671701	0.418078
C	-2.377202	0.183517	-1.387826
C	-4.842249	-0.642481	-0.390845
C	-3.500954	0.214500	-2.206292
H	-1.413427	0.490540	-1.799264
H	-5.799311	-0.972156	0.014381
H	-3.408984	0.553211	-3.239028
C	-0.521836	2.016395	0.028227
C	2.140355	-2.180414	-0.319385
O	3.210976	-2.328817	0.445423
O	-1.519977	2.579698	0.693783
O	2.072000	-2.549627	-1.463603
O	-0.002467	2.513204	-0.941397
C	4.360806	-2.902521	-0.179502
H	5.140774	-2.929129	0.587140
H	4.133963	-3.916924	-0.532314
H	4.676230	-2.278444	-1.026743
C	-2.019930	3.811849	0.173883
H	-2.399732	3.662669	-0.846253
H	-2.831256	4.116630	0.841552
H	-1.226302	4.570447	0.163363
N	-0.648720	-2.273052	-0.930257
Cl	-3.899304	-1.255629	2.052078
C	-4.737434	-0.194884	-1.706264
H	-5.625709	-0.173104	-2.339431

46

TS-CD1-3’ SCF Done: -1643.90416225 A.U.

C	1.580003	1.157541	-0.765674
C	-0.980222	0.345755	-1.106639
C	0.063690	-0.746968	-0.780519
C	1.228718	-0.098229	-0.027177
H	2.122029	1.070721	-1.709274
H	-1.533963	0.044620	-2.003383
H	0.413695	-1.167308	-1.735446
H	0.886171	0.172794	0.980827
N	-0.173992	1.534202	-1.568088
C	2.425092	-1.024092	0.121434
C	2.876755	-1.382307	1.395195
C	3.085375	-1.532918	-1.004004
C	3.969868	-2.236891	1.543747

H	2.361984	-0.991300	2.275891
C	4.176931	-2.387154	-0.856272
H	2.744585	-1.258885	-2.006193
C	4.621961	-2.741504	0.418849
H	4.311223	-2.510403	2.543882
H	4.682613	-2.777851	-1.741245
H	5.476607	-3.410625	0.534306
C	-1.963473	0.626887	0.010780
C	-3.271503	0.118671	-0.030787
C	-1.593986	1.353857	1.152224
C	-4.163670	0.296459	1.027128
C	-2.476881	1.543761	2.211451
H	-0.601403	1.802054	1.213402
H	-5.169770	-0.117245	0.953455
H	-2.156612	2.115601	3.083244
C	-0.578055	-1.855035	0.023373
C	1.869408	2.379541	0.009048
O	2.313437	3.361061	-0.766622
O	-1.418719	-2.563500	-0.713846
O	1.649386	2.514383	1.194675
O	-0.393041	-2.045571	1.200638
C	2.495249	4.630167	-0.140685
H	2.857404	5.305697	-0.921440
H	1.539099	4.990020	0.263314
H	3.231054	4.553818	0.670775
C	-2.205014	-3.539449	-0.031079
H	-2.793020	-3.060294	0.764321
H	-2.866353	-3.979828	-0.783309
H	-1.556636	-4.310034	0.406718
N	-0.583800	2.671004	-1.394311
C	-3.761782	1.006155	2.153833
H	-4.460059	1.145793	2.980464
Cl	-3.869611	-0.745309	-1.429272

46
D1-3 SCF Done: -1643.93207916 A.U.

C	-0.943097	-1.479449	-0.415552
C	1.504418	0.088283	-0.989266
C	0.191978	0.782389	-0.748538
C	-0.852654	-0.015503	0.069100
H	-0.988408	-1.546402	-1.508069
H	1.648229	-0.348902	-1.975527
H	-0.235508	0.968790	-1.746337
H	-0.540325	-0.054176	1.124487
N	0.242539	-2.309046	0.030946
C	-2.233285	0.619281	0.012399
C	-2.934740	0.857998	1.199400
C	-2.858807	0.892110	-1.210258
C	-4.233495	1.365720	1.168108
H	-2.454067	0.641957	2.156610
C	-4.158651	1.396631	-1.243061
H	-2.339706	0.695105	-2.151724
C	-4.849370	1.635935	-0.054361
H	-4.765138	1.551198	2.103261
H	-4.634792	1.601175	-2.203761
H	-5.866333	2.031494	-0.081086
C	2.550845	-0.045943	-0.036516
C	3.768882	-0.710670	-0.356718

C	2.440387	0.437312	1.296336
C	4.793125	-0.868948	0.565264
C	3.462660	0.281474	2.220477
H	1.523328	0.934397	1.615032
H	5.706187	-1.385052	0.266575
H	3.334579	0.666632	3.233086
C	0.383337	2.167759	-0.144170
C	-2.179632	-2.123236	0.194004
O	-3.156043	-2.236405	-0.686758
O	1.268387	2.876900	-0.831883
O	-2.265536	-2.431465	1.354901
O	-0.181864	2.589402	0.835374
C	-4.424603	-2.653658	-0.176243
H	-5.108743	-2.654816	-1.029839
H	-4.346680	-3.659671	0.256019
H	-4.767826	-1.946188	0.591464
C	1.556937	4.185042	-0.338325
H	1.966530	4.121133	0.678895
H	2.297977	4.610839	-1.021417
H	0.646529	4.798689	-0.329994
N	0.639350	-2.324791	1.126497
C	4.645417	-0.370362	1.861165
H	5.452422	-0.496937	2.583985
Cl	4.011827	-1.360755	-1.957430

46

D1-3’ SCF Done: -1643.93090174 A.U.

C	1.744692	0.988693	-0.893309
C	-1.302759	0.359745	-1.307666
C	-0.172584	-0.647095	-1.000900
C	0.991393	-0.079960	-0.176810
H	2.010230	0.881666	-1.946174
H	-2.065346	-0.184648	-1.880923
H	0.181381	-1.011677	-1.975644
H	0.593401	0.334421	0.760248
N	-0.777323	1.344614	-2.337445
C	1.998982	-1.162095	0.212985
C	2.478745	-1.212308	1.526351
C	2.476294	-2.085746	-0.725054
C	3.411202	-2.179008	1.901114
H	2.113046	-0.487238	2.257449
C	3.406997	-3.054452	-0.347916
H	2.124275	-2.053897	-1.758931
C	3.875738	-3.104344	0.965300
H	3.773026	-2.212078	2.930453
H	3.768397	-3.772805	-1.086110
H	4.602747	-3.863546	1.259238
C	-1.933455	1.005451	-0.097299
C	-3.108004	0.489591	0.467904
C	-1.352034	2.115366	0.531021
C	-3.685534	1.045474	1.607708
C	-1.914910	2.681854	1.671158
H	-0.437808	2.548243	0.121592
H	-4.599323	0.610859	2.013480
H	-1.434730	3.542670	2.138030
C	-0.789298	-1.818772	-0.258478
C	2.300929	2.100979	-0.142575
O	3.063277	2.901710	-0.893026

O	-1.262800	-2.739990	-1.084118
O	2.099711	2.302409	1.042805
O	-0.864275	-1.894206	0.943753
C	3.635940	4.025332	-0.232098
H	4.220636	4.559302	-0.987658
H	2.847915	4.677566	0.169062
H	4.286817	3.696680	0.589718
C	-1.902658	-3.864541	-0.479468
H	-2.736081	-3.531598	0.153302
H	-2.269189	-4.485282	-1.302513
H	-1.180590	-4.423894	0.130312
N	-1.033393	2.477493	-2.396850
C	-3.083900	2.146500	2.210229
H	-3.533303	2.583932	3.102992
Cl	-3.901909	-0.895694	-0.248020

46

TS-D1D-3 SCF Done: -1643.92991753 A.U.

C	-1.039494	-1.366371	-0.441204
C	1.459482	0.138015	-0.997431
C	0.153822	0.847987	-0.764726
C	-0.894322	0.061333	0.060342
H	-1.010441	-1.494578	-1.526390
H	1.604299	-0.301935	-1.982256
H	-0.277439	1.025122	-1.763103
H	-0.580456	0.018261	1.115946
N	0.343824	-2.296358	0.081227
C	-2.266396	0.722166	0.005749
C	-2.903147	1.133359	1.181630
C	-2.935514	0.869414	-1.215137
C	-4.178865	1.692953	1.137775
H	-2.388015	1.014415	2.137528
C	-4.212724	1.430321	-1.260582
H	-2.459523	0.541783	-2.143262
C	-4.837095	1.844242	-0.084230
H	-4.662059	2.012367	2.063042
H	-4.721484	1.540700	-2.219972
H	-5.836360	2.282145	-0.118219
C	2.495395	-0.014771	-0.036374
C	3.696188	-0.717217	-0.342222
C	2.387309	0.477106	1.293662
C	4.706935	-0.901531	0.589622
C	3.396143	0.294733	2.227698
H	1.482880	1.002694	1.601695
H	5.606578	-1.446441	0.301540
H	3.270177	0.687706	3.237571
C	0.365256	2.238664	-0.180872
C	-2.160288	-2.110595	0.212846
O	-2.869979	-2.823985	-0.649132
O	1.228875	2.937827	-0.906041
O	-2.372748	-2.078139	1.402260
O	-0.163412	2.672982	0.813398
C	-3.957225	-3.572274	-0.102785
H	-4.425953	-4.087227	-0.946466
H	-3.585687	-4.298498	0.632206
H	-4.674752	-2.895268	0.379783
C	1.539186	4.249637	-0.436300
H	1.979573	4.195336	0.568520

H	2.260883	4.663965	-1.146622
H	0.632619	4.868376	-0.407018
N	0.620315	-2.335292	1.183248
C	4.562523	-0.393122	1.882128
H	5.358815	-0.540438	2.612815
Cl	3.931322	-1.387101	-1.935888

46
TS-D1D-3’ SCF Done: -1643.92844875 A.U.

C	1.785568	0.965759	-0.870230
C	-1.290320	0.303745	-1.184742
C	-0.120720	-0.650867	-0.964909
C	1.045471	-0.100243	-0.134078
H	2.093857	0.805565	-1.904769
H	-2.001167	-0.146553	-1.884735
H	0.225814	-0.959679	-1.961980
H	0.654171	0.317142	0.805062
N	-0.671414	1.562411	-2.296752
C	2.071150	-1.169798	0.248253
C	2.850732	-0.962682	1.393830
C	2.294614	-2.313547	-0.527195
C	3.826164	-1.885688	1.765046
H	2.684081	-0.065791	1.996121
C	3.270249	-3.239498	-0.151794
H	1.707225	-2.504126	-1.428236
C	4.038196	-3.029276	0.992570
H	4.420742	-1.712974	2.664041
H	3.428528	-4.131010	-0.761480
H	4.799709	-3.754997	1.283478
C	-1.964889	0.905221	-0.004438
C	-3.204873	0.412418	0.439986
C	-1.409280	1.975907	0.713246
C	-3.865375	0.951716	1.540076
C	-2.058614	2.526158	1.815608
H	-0.449799	2.391928	0.404809
H	-4.821312	0.530989	1.853292
H	-1.597844	3.356927	2.351521
C	-0.726172	-1.862374	-0.273718
C	2.237324	2.167719	-0.194069
O	2.948972	2.969659	-0.991590
O	-1.158623	-2.769371	-1.137690
O	1.997622	2.441964	0.970277
O	-0.843656	-1.966093	0.922999
C	3.415322	4.182612	-0.409539
H	3.971897	4.704053	-1.194467
H	2.568801	4.796691	-0.072473
H	4.072008	3.970639	0.445326
C	-1.808082	-3.913430	-0.581472
H	-2.673195	-3.602659	0.019433
H	-2.129915	-4.523487	-1.430791
H	-1.105678	-4.474841	0.049136
N	-0.905728	2.661766	-2.158283
C	-3.288295	2.016648	2.228305
H	-3.803489	2.442620	3.090585
Cl	-3.949881	-0.947174	-0.370295

44
D-3 SCF Done: -1534.44958361 A.U.

C	-0.603568	-0.856652	-1.185610
C	0.126561	1.016588	0.926902
C	-0.657551	-0.214394	1.276514
C	-1.427634	-0.839083	0.059784
H	-0.547423	0.019711	-1.832230
H	-0.360065	1.971331	1.114700
H	-1.429944	0.088659	2.002914
H	-1.651662	-1.877440	0.343109
C	-2.739099	-0.104177	-0.164743
C	-3.944525	-0.718693	0.190147
C	-2.772617	1.191928	-0.697844
C	-5.160174	-0.054408	0.020926
H	-3.929085	-1.730388	0.602475
C	-3.986856	1.857200	-0.865149
H	-1.844389	1.693046	-0.985109
C	-5.184573	1.236174	-0.506745
H	-6.091492	-0.550421	0.301010
H	-3.996520	2.866797	-1.280244
H	-6.134326	1.757110	-0.641091
C	1.408128	1.033305	0.306864
C	1.998901	2.252066	-0.135904
C	2.183520	-0.140867	0.082347
C	3.243772	2.299597	-0.747854
C	3.425271	-0.098153	-0.530701
H	1.798867	-1.115324	0.389993
H	3.643082	3.261330	-1.071840
H	3.980693	-1.024961	-0.682750
C	0.136569	-1.258325	2.049587
C	0.199023	-2.007747	-1.557658
O	0.993971	-1.749245	-2.603265
O	0.963394	-0.693106	2.920893
O	0.193186	-3.084547	-0.988677
O	0.013232	-2.453185	1.939725
C	1.860161	-2.802825	-3.008196
H	2.450785	-2.410604	-3.842179
H	2.520354	-3.097588	-2.180399
H	1.278642	-3.676734	-3.332981
C	1.750851	-1.578100	3.716516
H	2.396902	-2.190422	3.072360
H	2.357543	-0.942270	4.368367
H	1.103416	-2.232796	4.314615
C	3.965396	1.122388	-0.949336
H	4.942369	1.162159	-1.432433
Cl	1.133686	3.756148	0.055483

46
TS-D1G-3 SCF Done: -1643.90304684 A.U.

C	-1.362832	-1.327546	-1.058768
C	1.531548	-0.095984	-0.897404
C	0.195870	0.645954	-0.908982
C	-0.952518	-0.104999	-0.197227
H	-2.001862	-1.024479	-1.896124
H	2.045681	-0.093649	-1.858087
H	-0.072878	0.775479	-1.969171
H	-0.577113	-0.505012	0.756361
N	-0.144530	-1.947888	-1.626483
C	-2.204508	0.698763	0.077050
C	-2.909459	0.480341	1.265283

C	-2.722691	1.594857	-0.865935
C	-4.108618	1.149593	1.512989
H	-2.510133	-0.222087	2.002612
C	-3.916964	2.268935	-0.616183
H	-2.189520	1.773892	-1.803664
C	-4.613990	2.047630	0.573382
H	-4.646432	0.970559	2.445870
H	-4.306813	2.970523	-1.356023
H	-5.549568	2.575555	0.766568
C	2.403681	-0.145866	0.267658
C	3.720373	-0.647701	0.149089
C	1.982778	0.220753	1.563387
C	4.575973	-0.748015	1.239702
C	2.831405	0.122919	2.660887
H	0.974377	0.603921	1.716366
H	5.585507	-1.134946	1.096391
H	2.472357	0.423739	3.645993
C	0.425941	2.054390	-0.378978
C	-2.108571	-2.319442	-0.182154
O	-3.400363	-2.354076	-0.458012
O	1.238578	2.728428	-1.184190
O	-1.581302	-2.960630	0.691645
O	-0.023178	2.513359	0.641384
C	-4.205578	-3.185531	0.380971
H	-5.231199	-3.082602	0.014827
H	-3.873726	-4.229328	0.307787
H	-4.134383	-2.847819	1.423692
C	1.604376	4.043101	-0.763201
H	2.113976	3.999540	0.208706
H	2.279977	4.430797	-1.531446
H	0.712313	4.678120	-0.682462
N	0.870694	-1.958985	-0.986666
C	4.130913	-0.357892	2.502509
H	4.802314	-0.436453	3.358838
Cl	4.315961	-1.179990	-1.401533

46

TS-D1G-3’ SCF Done: -1643.90856169 A.U.

C	-2.034905	-0.734303	-0.935446
C	1.109030	-0.868093	-1.203219
C	0.229964	0.407790	-1.012647
C	-1.030139	0.093416	-0.173188
H	-2.626975	-0.244658	-1.710800
H	1.767444	-0.714787	-2.070600
H	-0.060454	0.747737	-2.015706
H	-0.713547	-0.477852	0.711495
N	-0.846101	-1.751492	-2.138860
C	-1.754718	1.335817	0.323842
C	-2.306810	1.334048	1.609391
C	-1.916693	2.465058	-0.488665
C	-3.000490	2.447325	2.082465
H	-2.186580	0.449989	2.240873
C	-2.606168	3.580361	-0.012113
H	-1.499467	2.481723	-1.498733
C	-3.149363	3.574423	1.273438
H	-3.422148	2.435500	3.089208
H	-2.720458	4.458218	-0.650692
H	-3.688221	4.448165	1.644247

C	1.931499	-1.280085	-0.004371
C	3.224979	-0.781688	0.196145
C	1.421232	-2.159142	0.958086
C	3.992173	-1.140126	1.300546
C	2.173490	-2.524805	2.072767
H	0.415015	-2.561934	0.825360
H	4.996096	-0.730533	1.415169
H	1.750290	-3.210406	2.808068
C	1.021590	1.513230	-0.347768
C	-2.664264	-1.865980	-0.242859
O	-3.771768	-2.285139	-0.851391
O	1.401472	2.449182	-1.205572
O	-2.202643	-2.403726	0.745524
O	1.269543	1.547682	0.833319
C	-4.402602	-3.433570	-0.290857
H	-5.282476	-3.631668	-0.910606
H	-3.719340	-4.293563	-0.313526
H	-4.704599	-3.236156	0.746597
C	2.168516	3.525001	-0.665478
H	3.089806	3.138803	-0.208503
H	2.405275	4.182895	-1.506988
H	1.581338	4.064287	0.090103
N	0.164467	-1.969693	-1.546933
C	3.460018	-2.017204	2.243275
H	4.057133	-2.300602	3.111371
Cl	3.915437	0.338254	-0.957111

46

G SCF Done: -1643.99718604 A.U.

C	-1.464844	-1.058249	-1.242893
C	1.332317	-0.567734	-1.315431
C	0.340036	0.563219	-0.976848
C	-0.855006	-0.054683	-0.258357
H	-2.027365	-0.527678	-2.026514
H	2.042295	-0.199728	-2.067256
H	0.026865	1.013182	-1.929166
H	-0.483230	-0.609089	0.617773
N	-0.512260	-1.938251	-1.952675
C	-1.894641	0.932116	0.229092
C	-2.607334	0.645499	1.399805
C	-2.193934	2.102542	-0.478176
C	-3.602947	1.507780	1.855910
H	-2.371015	-0.265028	1.958449
C	-3.186456	2.969712	-0.018051
H	-1.651229	2.350319	-1.393820
C	-3.894257	2.674250	1.146925
H	-4.148657	1.270875	2.771136
H	-3.407268	3.881625	-0.575897
H	-4.670086	3.353829	1.504111
C	2.128285	-1.097160	-0.136977
C	3.346971	-0.514469	0.238333
C	1.666469	-2.173369	0.630875
C	4.080070	-0.971116	1.329385
C	2.387312	-2.642612	1.727991
H	0.720519	-2.653400	0.370291
H	5.022181	-0.486005	1.586435
H	2.000621	-3.481524	2.307968
C	0.966202	1.644239	-0.125563

C	-2.408150	-1.981938	-0.499809
O	-3.676749	-1.649677	-0.656986
O	1.310024	2.711353	-0.832084
O	-2.026644	-2.885051	0.204623
O	1.126494	1.552326	1.067956
C	-4.629365	-2.393401	0.106205
H	-5.608457	-1.970389	-0.136868
H	-4.589387	-3.454688	-0.171392
H	-4.419707	-2.284678	1.178905
C	1.938296	3.770181	-0.110045
H	2.857536	3.406209	0.368754
H	2.171094	4.545268	-0.846336
H	1.255822	4.162080	0.655967
N	0.694977	-1.727296	-1.982312
C	3.594855	-2.042362	2.076878
H	4.166770	-2.403885	2.932810
Cl	3.982670	0.841486	-0.664941

46

TS-CD SCF Done: -1643.93662738 A.U.

C	0.614385	1.935236	-0.341888
C	-0.881093	-0.800442	-0.862631
C	0.546517	-0.705788	-0.627370
C	1.159059	0.704999	0.165413
H	1.052971	2.436483	-1.206092
H	-1.147563	-1.008626	-1.897568
H	1.051067	-0.701327	-1.598761
H	0.858879	0.555765	1.210271
N	-1.199503	1.210687	-1.383631
C	2.656631	0.539643	0.024647
C	3.404326	0.109670	1.125881
C	3.306570	0.748616	-1.198326
C	4.779261	-0.106192	1.008956
H	2.901495	-0.066699	2.079991
C	4.678238	0.534869	-1.314312
H	2.734612	1.073451	-2.071306
C	5.419368	0.104287	-0.211053
H	5.349207	-0.441592	1.877568
H	5.172033	0.700860	-2.273619
H	6.493249	-0.066784	-0.304837
C	-1.944478	-1.003212	0.103973
C	-3.253743	-1.313902	-0.337523
C	-1.769984	-0.809704	1.491137
C	-4.314399	-1.459085	0.548431
C	-2.826696	-0.946775	2.381628
H	-0.785331	-0.549035	1.870520
H	-5.304481	-1.707197	0.165302
H	-2.656623	-0.789758	3.447385
C	1.150588	-1.819406	0.198218
C	-0.289288	2.718293	0.479046
O	-0.630611	3.871419	-0.117762
O	2.056489	-2.486136	-0.506991
O	-0.755890	2.388308	1.557667
O	0.898827	-2.069228	1.351747
C	-1.593563	4.669462	0.558587
H	-1.752957	5.554214	-0.066383
H	-2.536107	4.116324	0.677242
H	-1.224421	4.969208	1.549061

C	2.770959	-3.508153	0.187936
H	2.079840	-4.287255	0.535725
H	3.482816	-3.922669	-0.532272
H	3.303098	-3.078594	1.048095
N	-2.037405	1.774109	-1.837453
Cl	-3.594845	-1.518813	-2.036167
C	-4.099069	-1.277063	1.913651
H	-4.933127	-1.387513	2.608406

2
N2 SCF Done: -109.514336598 A.U.

N	0.000000	0.000000	0.546529
N	0.000000	0.000000	-0.546529

44
D-bs SCF Done: -1534.45348914 A.U.

C	-0.319433	1.238287	0.930082
C	-0.020404	-1.123387	-1.015190
C	-0.433212	0.244559	-1.433381
C	-1.145534	1.080906	-0.290688
H	-0.220086	0.426446	1.650818
H	-0.677828	-1.946597	-1.286772
H	-1.191430	0.137981	-2.226460
H	-1.307563	2.077952	-0.723447
C	-2.496965	0.454308	0.013932
C	-3.660175	1.048286	-0.487324
C	-2.608132	-0.726763	0.759642
C	-4.910205	0.472967	-0.255861
H	-3.583335	1.971713	-1.066530
C	-3.857318	-1.303070	0.990627
H	-1.713816	-1.207154	1.164202
C	-5.012068	-0.705455	0.483379
H	-5.807697	0.949736	-0.654563
H	-3.927336	-2.224326	1.572062
H	-5.989075	-1.156459	0.666518
C	1.089209	-1.397281	-0.174724
C	1.302448	-2.691400	0.387871
C	2.033409	-0.398720	0.200687
C	2.347574	-2.961050	1.255744
C	3.075871	-0.663329	1.079169
H	1.958405	0.608171	-0.210045
H	2.459426	-3.966666	1.662463
H	3.768767	0.136377	1.344949
C	0.668802	1.061421	-2.091931
C	0.366375	2.480205	1.241408
O	1.215904	2.336835	2.269489
O	1.431814	0.312621	-2.877794
O	0.219098	3.539078	0.659984
O	0.825937	2.250521	-1.959436
C	1.940522	3.500256	2.651615
H	2.575011	3.203419	3.492840
H	2.560072	3.858954	1.817962
H	1.252732	4.299783	2.959916
C	2.493818	0.980640	-3.558382
H	3.189353	1.421410	-2.831050
H	3.001212	0.215753	-4.153931
H	2.094155	1.769942	-4.208540
C	3.239699	-1.943297	1.610177

H	4.059208	-2.159944	2.296596
Cl	0.199536	-3.989260	0.012586

44
TS-DD’ SCF Done: -1534.44646071 A.U.

C	0.686234	-1.694406	0.376282
C	-0.913037	0.701904	-1.090027
C	-0.366997	-0.570717	-1.660403
C	0.940510	-1.070864	-0.953081
H	-0.224288	-2.269349	0.535560
H	-0.604860	1.613367	-1.597845
H	-0.058107	-0.337627	-2.693263
H	1.318381	-1.878761	-1.608121
C	1.959295	0.055666	-0.957437
C	2.908395	0.137204	-1.978390
C	1.908772	1.073798	0.004243
C	3.796533	1.213939	-2.039865
H	2.953618	-0.651787	-2.733537
C	2.790157	2.150319	-0.056924
H	1.173380	1.019021	0.812233
C	3.737587	2.224119	-1.081344
H	4.535838	1.261240	-2.841739
H	2.735408	2.937746	0.697390
H	4.428973	3.067518	-1.128962
C	-1.618444	0.849749	0.136911
C	-1.758796	2.128480	0.753032
C	-2.213613	-0.236455	0.835233
C	-2.430994	2.308312	1.952605
C	-2.886818	-0.060928	2.035975
H	-2.175029	-1.240240	0.412822
H	-2.500648	3.308467	2.381829
H	-3.333043	-0.924219	2.531695
C	-1.363014	-1.700334	-1.851291
C	1.695089	-1.834913	1.415062
O	1.179720	-2.387919	2.524332
O	-2.571083	-1.255778	-2.170757
O	2.867512	-1.522607	1.331728
O	-1.090185	-2.874257	-1.772582
C	2.084020	-2.581802	3.605021
H	1.502116	-3.034201	4.414349
H	2.903348	-3.250942	3.307697
H	2.504823	-1.621006	3.932658
C	-3.572676	-2.245558	-2.405916
H	-3.719993	-2.855923	-1.504540
H	-4.488602	-1.698626	-2.649019
H	-3.279514	-2.892591	-3.243151
C	-3.000945	1.210774	2.601820
H	-3.529331	1.355163	3.545051
Cl	-1.030454	3.533384	0.016358

44
D’-bs SCF Done: -1534.45328677 A.U.

C	-0.671510	0.349453	1.712899
C	0.437882	0.607359	-1.152642
C	-0.277224	1.687958	-0.413655
C	-1.312076	1.191113	0.664201
H	-0.286205	0.807210	2.623792
H	0.029008	0.317050	-2.118018

H	-0.859449	2.265453	-1.150090
H	-1.630039	2.117472	1.166451
C	-2.567478	0.557799	0.072017
C	-3.529194	0.061542	0.965555
C	-2.835124	0.481155	-1.297639
C	-4.717269	-0.499612	0.506130
H	-3.340103	0.118055	2.041086
C	-4.027901	-0.080945	-1.761197
H	-2.121214	0.854928	-2.032295
C	-4.971951	-0.575328	-0.864967
H	-5.448616	-0.879626	1.222061
H	-4.212725	-0.132549	-2.835863
H	-5.901013	-1.016649	-1.230058
C	1.531298	-0.143818	-0.644995
C	2.013230	-1.302398	-1.324925
C	2.173504	0.157204	0.588083
C	3.035542	-2.088798	-0.821381
C	3.192396	-0.636555	1.102020
H	1.869718	1.029068	1.167055
H	3.360704	-2.966823	-1.380638
H	3.650023	-0.370047	2.055859
C	0.642446	2.730881	0.205645
C	-0.454748	-1.076679	1.530355
O	0.212548	-1.626609	2.555027
O	1.610840	3.084029	-0.627656
O	-0.802727	-1.720594	0.557018
O	0.510049	3.211464	1.305573
C	0.538499	-3.004624	2.417785
H	1.121511	-3.272371	3.304906
H	-0.374458	-3.614403	2.366777
H	1.133009	-3.170129	1.507701
C	2.539501	4.057493	-0.150448
H	3.058818	3.678051	0.740290
H	3.251848	4.221892	-0.964428
H	2.018926	4.991663	0.098028
C	3.631245	-1.759741	0.401535
H	4.433813	-2.384149	0.796338
Cl	1.280844	-1.791833	-2.829700

44
E SCF Done: -1534.53200647 A.U.

C	0.653059	-1.077976	0.410606
C	-0.390474	0.000057	0.853918
C	0.653635	1.077823	0.411410
C	1.535162	-0.000064	-0.253132
H	1.119497	-1.537618	1.293784
H	-0.551597	-0.000285	1.933579
H	1.120341	1.536445	1.294981
H	1.371682	0.000403	-1.340229
C	3.012893	-0.000575	0.028456
C	3.936894	0.000039	-1.021657
C	3.491429	-0.001662	1.346434
C	5.309131	-0.000434	-0.764629
H	3.575033	0.000898	-2.052567
C	4.860545	-0.002142	1.606003
H	2.783832	-0.002156	2.180759
C	5.774556	-0.001531	0.549593
H	6.016229	0.000051	-1.596454

H	5.217025	-0.003002	2.637847
H	6.847094	-0.001912	0.752668
C	-1.717449	0.000474	0.138640
C	-2.940030	-0.000431	0.826434
C	-1.784889	0.001463	-1.265671
C	-4.166692	-0.000273	0.161545
C	-3.000499	0.001643	-1.945029
H	-0.863164	0.002045	-1.852011
H	-5.089666	-0.000983	0.742243
H	-3.008907	0.002436	-3.035899
C	0.151073	2.181824	-0.475927
C	0.149615	-2.180844	-0.477637
O	-0.780470	-2.907048	0.132468
O	-0.778727	2.908031	0.134602
O	0.508702	-2.382297	-1.612955
O	0.510676	2.384174	-1.610922
C	-1.406528	-3.924764	-0.647166
H	-2.136589	-4.407263	0.009847
H	-1.911624	-3.477021	-1.514582
H	-0.662820	-4.654540	-0.993407
C	-1.403694	3.927097	-0.644144
H	-1.908856	3.480715	-1.512220
H	-2.133591	4.409471	0.013144
H	-0.659272	4.656645	-0.989330
C	-4.197138	0.000790	-1.229405
H	-5.156229	0.000922	-1.749354
Cl	-2.986674	-0.001836	2.575283

44

E’ SCF Done: -1534.53285466 A.U.

C	-0.249804	-0.947567	0.281525
C	0.853153	-0.224448	-0.552884
C	-0.076799	1.010960	-0.603111
C	-0.881702	0.456336	0.577698
H	0.084834	-1.520475	1.153883
H	1.015464	-0.679487	-1.536783
H	-0.664175	0.937468	-1.532406
H	-0.452613	0.824825	1.523360
C	-2.379449	0.601535	0.596724
C	-3.160505	-0.281643	1.355310
C	-3.014422	1.615624	-0.127572
C	-4.548924	-0.158229	1.378969
H	-2.675008	-1.076443	1.927141
C	-4.404343	1.743313	-0.098890
H	-2.419139	2.319123	-0.714453
C	-5.175183	0.855240	0.650440
H	-5.145090	-0.855861	1.970427
H	-4.885793	2.540473	-0.668460
H	-6.262273	0.951889	0.667968
C	2.160150	-0.156980	0.193834
C	3.036700	-1.252281	0.122727
C	2.546934	0.905881	1.020090
C	4.256479	-1.280233	0.792374
C	3.762864	0.894790	1.703431
H	1.884723	1.761069	1.163310
H	4.904075	-2.152482	0.697210
H	4.031313	1.742412	2.335536
C	0.452156	2.411449	-0.543226

C	-1.155679	-1.783519	-0.577514
O	-1.675879	-2.811245	0.081885
O	1.443740	2.603512	-1.403842
O	-1.424931	-1.535866	-1.730822
O	0.024513	3.277147	0.185118
C	-2.632184	-3.600756	-0.625108
H	-2.952321	-4.384595	0.067921
H	-3.488102	-2.979411	-0.922519
H	-2.173501	-4.044595	-1.518526
C	2.036642	3.901572	-1.417587
H	2.482334	4.123819	-0.438011
H	2.811996	3.874615	-2.189171
H	1.283279	4.663135	-1.658086
C	4.624499	-0.192809	1.582182
H	5.579395	-0.206594	2.109699
Cl	2.576327	-2.659805	-0.809013

44
TS-DF SCF Done: -1534.44470021 A.U.

C	-0.606598	2.257805	-0.326523
C	1.128046	-0.204638	0.952323
C	-0.291254	0.051979	1.024124
C	-0.911891	0.862519	-0.463220
H	-1.358969	2.994258	-0.043361
H	1.742507	0.411470	1.605856
H	-0.512418	0.775838	1.812708
H	-0.274334	0.362178	-1.203764
C	-2.360270	0.509603	-0.651403
C	-2.705597	-0.516064	-1.539345
C	-3.376217	1.151405	0.070373
C	-4.040069	-0.884766	-1.716794
H	-1.917971	-1.026229	-2.099797
C	-4.707930	0.780709	-0.102486
H	-3.124461	1.936298	0.787455
C	-5.044556	-0.236848	-0.998864
H	-4.292997	-1.681273	-2.418893
H	-5.488868	1.288168	0.466922
H	-6.088785	-0.524662	-1.134000
C	1.839784	-1.073010	0.062934
C	3.256354	-1.177920	0.144175
C	1.222148	-1.794779	-0.989585
C	3.994075	-1.960865	-0.731876
C	1.957498	-2.572805	-1.874532
H	0.141395	-1.747676	-1.099154
H	5.077536	-2.013794	-0.620836
H	1.442634	-3.111930	-2.670924
C	-1.211160	-1.126930	1.230413
C	0.767213	2.668013	-0.404835
O	0.964509	3.976867	-0.170500
O	-2.186840	-0.805159	2.073326
O	1.695616	1.900173	-0.629435
O	-1.124968	-2.216598	0.718526
C	2.311815	4.425275	-0.236834
H	2.289044	5.500232	-0.030180
H	2.929940	3.910184	0.511901
H	2.732891	4.242025	-1.235591
C	-3.217441	-1.775972	2.246774
H	-2.808765	-2.692050	2.693688

H	-3.954022	-1.321643	2.916768
H	-3.676290	-2.014655	1.276288
C	3.343707	-2.665141	-1.746729
H	3.926296	-3.278910	-2.435287
Cl	4.137834	-0.303265	1.370372

44
TS-DDtw SCF Done: -1534.44427497 A.U.

C	2.104292	1.412670	0.186227
C	-0.680131	-0.895461	-0.976519
C	0.755170	-0.477827	-0.998658
C	1.001433	1.107905	-0.748918
H	1.975199	1.995514	1.096582
H	-1.123216	-1.099779	-1.949278
H	1.108602	-0.673924	-2.015605
H	1.350383	1.423176	-1.746168
C	-0.291878	1.851636	-0.474241
C	-1.194091	2.013506	-1.535338
C	-0.665642	2.303043	0.793593
C	-2.450066	2.579090	-1.330170
H	-0.909194	1.671732	-2.534229
C	-1.921537	2.879756	1.002288
H	0.006276	2.185594	1.645326
C	-2.821017	3.009239	-0.053806
H	-3.142038	2.684466	-2.167918
H	-2.198928	3.217577	2.002708
H	-3.805634	3.449787	0.113372
C	-1.543931	-0.959992	0.154637
C	-2.935896	-1.221751	0.004217
C	-1.099782	-0.720725	1.483152
C	-3.806126	-1.264044	1.084483
C	-1.964857	-0.767035	2.565981
H	-0.049297	-0.485457	1.649455
H	-4.863877	-1.466651	0.912293
H	-1.580204	-0.578986	3.569630
C	1.613920	-1.389194	-0.133659
C	3.430604	0.915585	-0.120291
O	4.354333	1.290388	0.768290
O	1.962636	-2.478613	-0.806031
O	3.687800	0.202151	-1.077805
O	1.944817	-1.199358	1.013547
C	5.673326	0.800935	0.549808
H	6.289364	1.211288	1.356053
H	5.684607	-0.297431	0.585945
H	6.052190	1.135467	-0.425564
C	2.785762	-3.414241	-0.110883
H	2.280471	-3.768864	0.797077
H	2.954325	-4.244196	-0.803627
H	3.740684	-2.942446	0.159160
C	-3.321865	-1.040942	2.374803
H	-4.007737	-1.076023	3.222382
Cl	-3.609693	-1.481782	-1.585403

44
Dtw-bs SCF Done: -1534.45339963 A.U.

C	2.727514	0.771842	-0.298451
C	-0.559891	-1.207064	-0.675885
C	0.793897	-0.861050	-0.154670

C	1.327118	0.525115	-0.722677
H	2.998903	1.567335	0.393599
H	-0.572999	-1.729523	-1.631161
H	1.493059	-1.614491	-0.554075
H	1.340429	0.347419	-1.810279
C	0.371567	1.666429	-0.441237
C	-0.650011	1.931400	-1.362169
C	0.423562	2.417837	0.739159
C	-1.614485	2.903873	-1.102151
H	-0.698123	1.351044	-2.287650
C	-0.538242	3.395157	0.999587
H	1.204561	2.225486	1.476600
C	-1.564190	3.635833	0.085186
H	-2.407832	3.089518	-1.828784
H	-0.486954	3.968404	1.927265
H	-2.318920	4.396386	0.293900
C	-1.810180	-0.815070	-0.122664
C	-3.037203	-1.117370	-0.780808
C	-1.929765	-0.076511	1.088520
C	-4.269577	-0.714912	-0.284672
C	-3.157079	0.329832	1.586055
H	-1.032658	0.216311	1.634605
H	-5.175854	-0.969918	-0.835195
H	-3.196438	0.904594	2.512775
C	0.951714	-0.978614	1.349687
C	3.786157	-0.074850	-0.809480
O	4.991694	0.252477	-0.329036
O	0.359464	-2.069946	1.817971
O	3.620107	-0.995280	-1.593852
O	1.573520	-0.217652	2.052380
C	6.085927	-0.534408	-0.785516
H	6.977787	-0.133086	-0.293961
H	5.943438	-1.588588	-0.510198
H	6.187873	-0.456755	-1.876843
C	0.451282	-2.291797	3.224739
H	-0.021378	-1.462673	3.769227
H	-0.081956	-3.227043	3.420126
H	1.502310	-2.379094	3.530004
C	-4.336713	0.013266	0.903758
H	-5.305914	0.331906	1.289464
Cl	-3.024472	-2.006332	-2.283352

44
TS-DtwF SCF Done: -1534.44697881 A.U.

C	2.712947	0.843839	-0.234561
C	-0.495637	-1.209279	-0.712262
C	0.794235	-0.974200	-0.155362
C	1.386770	0.690468	-0.726724
H	2.955832	1.443845	0.640478
H	-0.503785	-1.582534	-1.736307
H	1.571734	-1.570448	-0.651100
H	1.366211	0.433570	-1.790770
C	0.341258	1.694614	-0.360308
C	-0.713772	1.919201	-1.255087
C	0.344169	2.376080	0.864268
C	-1.749546	2.795141	-0.934035
H	-0.728380	1.383034	-2.208163
C	-0.691098	3.253505	1.185889

H	1.147115	2.207158	1.583556
C	-1.742788	3.462151	0.291864
H	-2.565704	2.953858	-1.641563
H	-0.677813	3.774723	2.144992
H	-2.554063	4.145730	0.549272
C	-1.767422	-0.843757	-0.152644
C	-2.970692	-1.060169	-0.874302
C	-1.907707	-0.207421	1.107496
C	-4.213334	-0.678006	-0.382749
C	-3.142676	0.182092	1.601892
H	-1.022788	0.034788	1.696160
H	-5.105932	-0.866720	-0.980049
H	-3.200802	0.682375	2.569742
C	0.971209	-1.084161	1.343640
C	3.777988	0.070308	-0.838453
O	4.979616	0.328226	-0.303515
O	0.396254	-2.192429	1.799326
O	3.626298	-0.748044	-1.733078
O	1.574301	-0.322651	2.058428
C	6.078438	-0.393991	-0.845273
H	6.965587	-0.059063	-0.298287
H	5.935613	-1.474912	-0.707983
H	6.192173	-0.179179	-1.916997
C	0.489595	-2.424940	3.204676
H	0.000626	-1.609593	3.755704
H	-0.026498	-3.371831	3.389826
H	1.541360	-2.495488	3.511699
Cl	-2.930030	-1.809809	-2.450024
C	-4.303998	-0.053783	0.860126
H	-5.279358	0.250460	1.242353

44
F SCF Done: -1534.50817336 A.U.

C	-1.004977	-1.570804	-1.357141
C	0.240867	1.432149	0.930106
C	-0.325710	0.589063	1.809581
C	-1.907937	-1.284199	-0.405080
H	-0.986368	-1.101272	-2.342168
H	-0.320501	2.352409	0.743559
H	-1.306134	0.852223	2.213423
H	-1.815796	-1.815797	0.547915
C	-2.998640	-0.307960	-0.481196
C	-3.771638	-0.090253	0.670319
C	-3.284146	0.428818	-1.643018
C	-4.807205	0.842265	0.664922
H	-3.543049	-0.658191	1.575884
C	-4.320178	1.357113	-1.647414
H	-2.693821	0.273245	-2.548001
C	-5.083375	1.566853	-0.494669
H	-5.399477	1.003988	1.567193
H	-4.536298	1.923419	-2.555036
H	-5.894638	2.297182	-0.503223
C	1.470227	1.304021	0.126664
C	2.170902	2.454114	-0.279937
C	1.940828	0.069943	-0.354510
C	3.284362	2.387071	-1.114193
C	3.043962	-0.013050	-1.196499
H	1.427498	-0.839035	-0.047999

H	3.801091	3.304885	-1.396417
H	3.376361	-0.986750	-1.559403
C	0.162530	-0.717939	2.313998
C	0.064778	-2.540586	-1.055625
O	0.925115	-2.667873	-2.064561
O	1.482528	-0.836504	2.361193
O	0.197683	-3.138179	-0.006611
O	-0.591137	-1.591742	2.685394
C	2.013088	-3.565179	-1.858927
H	2.625680	-3.515969	-2.764537
H	2.602118	-3.262079	-0.981950
H	1.640544	-4.587366	-1.706876
C	1.995294	-2.105780	2.764244
H	1.643607	-2.887218	2.076608
H	3.085526	-2.019103	2.721484
H	1.671628	-2.341343	3.786915
C	3.718502	1.147866	-1.577035
H	4.585443	1.093226	-2.237292
Cl	1.665522	4.028633	0.277591

49

C-4-Me SCF Done: -1223.67999211 A.U.

C	1.344876	1.176250	-0.839732
C	-0.887085	0.307340	-1.493497
C	0.050583	-0.816471	-1.001529
C	1.067721	-0.116375	-0.085879
H	2.039678	1.034773	-1.678196
H	-1.152659	0.174446	-2.548620
H	0.560011	-1.261921	-1.868712
H	0.555009	0.142795	0.853542
N	-0.008780	1.555509	-1.425694
C	2.284937	-0.948132	0.241652
C	2.517372	-1.355594	1.559386
C	3.183495	-1.341313	-0.758932
C	3.629409	-2.137503	1.874993
H	1.815291	-1.059744	2.342353
C	4.294523	-2.122057	-0.444592
H	3.014669	-1.037544	-1.795451
C	4.520967	-2.521757	0.874043
H	3.797861	-2.447481	2.907994
H	4.987181	-2.420360	-1.233737
H	5.391596	-3.132692	1.119520
C	-2.135316	0.489990	-0.663765
C	-3.313381	-0.146163	-1.063341
C	-2.130503	1.204920	0.541801
C	-4.460268	-0.080813	-0.271004
C	-3.279634	1.270258	1.324374
H	-1.227276	1.726087	0.870216
H	-5.371780	-0.584576	-0.601274
H	-3.259150	1.834798	2.259832
C	-0.706237	-1.906971	-0.284222
C	1.797451	2.311173	0.046464
O	2.955238	2.820066	-0.332351
O	-1.392451	-2.662999	-1.131858
O	1.169280	2.696862	1.001971
O	-0.724301	-2.062016	0.912254
C	3.472513	3.885501	0.468396
H	4.428052	4.167545	0.016912

H	2.776688	4.734591	0.458389
H	3.620687	3.542202	1.500620
C	-2.233961	-3.661335	-0.554007
H	-2.972049	-3.193014	0.111807
H	-2.734762	-4.158996	-1.389899
H	-1.632148	-4.382239	0.014967
N	-0.344004	2.657797	-1.746296
C	-4.463056	0.626163	0.935665
H	-3.332494	-0.704871	-2.002182
C	-5.696040	0.712148	1.793466
H	-5.486634	0.368381	2.817363
H	-6.049430	1.752042	1.866873
H	-6.511165	0.101781	1.383049

49
TS-CD-4-Me SCF Done: -1223.64806848 A.U.

C	-0.691031	1.946069	0.229830
C	0.759478	-0.612800	1.410485
C	-0.617256	-0.601542	0.936870
C	-1.098863	0.615481	-0.157076
H	-1.285757	2.540776	0.924481
H	0.865180	-0.667168	2.495933
H	-1.278990	-0.473047	1.799874
H	-0.609642	0.329198	-1.096804
N	0.950310	1.461733	1.659547
C	-2.593937	0.402473	-0.274743
C	-3.112861	-0.225157	-1.412053
C	-3.468003	0.765172	0.758089
C	-4.481307	-0.483907	-1.518547
H	-2.435806	-0.522164	-2.216856
C	-4.833095	0.508378	0.651681
H	-3.076838	1.246545	1.658195
C	-5.344590	-0.119434	-0.486872
H	-4.870730	-0.974203	-2.412890
H	-5.502391	0.795996	1.464707
H	-6.413860	-0.323247	-0.567165
C	1.965021	-0.942080	0.679287
C	3.121334	-1.227792	1.432769
C	2.076342	-0.911531	-0.727863
C	4.332613	-1.500933	0.809985
C	3.292351	-1.177248	-1.338942
H	1.211110	-0.672168	-1.342484
H	5.213656	-1.727830	1.414095
H	3.362504	-1.145016	-2.428708
C	-1.028655	-1.866668	0.218521
C	0.313805	2.642794	-0.534968
O	0.521675	3.890388	-0.071047
O	-2.040805	-2.459330	0.841499
O	0.973419	2.187787	-1.459865
O	-0.549737	-2.284199	-0.807544
C	1.566587	4.626562	-0.690886
H	1.596766	5.599770	-0.189649
H	2.529452	4.110338	-0.567708
H	1.367030	4.762675	-1.762936
C	-2.584138	-3.614901	0.203573
H	-1.823287	-4.403266	0.130509
H	-3.416687	-3.944738	0.832433
H	-2.943305	-3.357395	-0.802511

N	1.687966	2.137655	2.133856
C	4.440045	-1.481737	-0.586927
C	5.740656	-1.779550	-1.275483
H	5.666222	-2.714732	-1.852198
H	5.997294	-0.981451	-1.987785
H	6.561407	-1.884653	-0.554372
H	3.055337	-1.236982	2.523295

50

C-4-OMe SCF Done: -1298.88416321 A.U.

C	1.666082	1.172466	-0.774551
C	-0.541167	0.317582	-1.536744
C	0.378852	-0.814176	-1.030478
C	1.360489	-0.134054	-0.063048
H	2.390428	1.055620	-1.591734
H	-0.761005	0.207352	-2.604933
H	0.919985	-1.244500	-1.886015
H	0.815534	0.105391	0.862904
N	0.329630	1.567120	-1.405199
C	2.563283	-0.975936	0.290956
C	2.768406	-1.378101	1.614739
C	3.476200	-1.382127	-0.691194
C	3.867881	-2.167654	1.954451
H	2.054986	-1.071634	2.383262
C	4.574730	-2.170516	-0.352931
H	3.328429	-1.081678	-1.731982
C	4.773895	-2.565041	0.971699
H	4.015104	-2.473689	2.991872
H	5.278805	-2.479167	-1.127888
H	5.634516	-3.182346	1.235841
C	-1.823202	0.484630	-0.758692
C	-2.989030	-0.117857	-1.232172
C	-1.867429	1.151795	0.476239
C	-4.179705	-0.077222	-0.503200
H	-2.972873	-0.639144	-2.192267
C	-3.044074	1.205587	1.208809
H	-0.976316	1.648713	0.869603
C	-4.208051	0.587120	0.727274
H	-5.070811	-0.556704	-0.906441
H	-3.088862	1.726502	2.166452
O	-5.306601	0.687624	1.508273
C	-6.504930	0.085335	1.063256
H	-6.382176	-1.002013	0.937944
H	-7.255457	0.277251	1.838256
H	-6.843638	0.526128	0.112415
C	-0.407600	-1.916149	-0.365114
C	2.063059	2.307012	0.138265
O	3.054659	3.027095	-0.351234
O	-1.078041	-2.640762	-1.252214
O	1.510174	2.540293	1.185668
O	-0.461216	-2.104224	0.825503
C	3.463707	4.154646	0.426497
H	4.286791	4.620083	-0.123298
H	2.627326	4.857591	0.534959
H	3.800702	3.825956	1.418242
C	-1.950258	-3.640651	-0.725113
H	-2.698789	-3.178857	-0.066401
H	-2.435922	-4.107160	-1.587547

H	-1.375715	-4.386821	-0.160504
N	0.000416	2.677562	-1.699715

50
TS-CD-4-OMe SCF Done: -1298.85375158 A.U.

C	-1.082793	1.935371	0.234924
C	0.528284	-0.488633	1.439696
C	-0.855663	-0.567692	0.964926
C	-1.378557	0.564306	-0.148009
H	-1.738709	2.485171	0.911227
H	0.637269	-0.553785	2.524288
H	-1.517751	-0.447911	1.829940
H	-0.843467	0.318449	-1.074159
N	0.548958	1.572071	1.695749
C	-2.851627	0.253440	-0.329239
C	-3.281079	-0.411327	-1.482537
C	-3.788792	0.556723	0.666702
C	-4.623138	-0.765034	-1.641004
H	-2.553996	-0.662821	-2.258638
C	-5.127496	0.204602	0.509255
H	-3.468231	1.067547	1.578369
C	-5.549244	-0.460083	-0.645020
H	-4.942150	-1.283355	-2.547394
H	-5.846418	0.446541	1.294321
H	-6.597791	-0.738279	-0.765587
C	1.745438	-0.733818	0.707019
C	2.932773	-0.872040	1.455210
C	1.845409	-0.755325	-0.705621
C	4.168328	-1.045653	0.848060
H	2.875846	-0.841938	2.545924
C	3.069272	-0.923799	-1.320295
H	0.956582	-0.645239	-1.322713
C	4.241369	-1.072438	-0.553548
H	5.061470	-1.153968	1.461668
H	3.157738	-0.941949	-2.407395
O	5.380758	-1.231036	-1.241102
C	6.597312	-1.372608	-0.530051
H	6.577760	-2.266135	0.112350
H	7.383168	-1.484133	-1.284846
H	6.801831	-0.481296	0.082244
C	-1.189662	-1.890592	0.310550
C	-0.124929	2.699657	-0.515389
O	-0.017228	3.964356	-0.055546
O	-2.189095	-2.497410	0.939519
O	0.590675	2.291910	-1.423458
O	-0.667037	-2.336169	-0.682016
C	0.987449	4.767862	-0.656187
H	0.940068	5.742972	-0.159630
H	1.981259	4.320618	-0.510560
H	0.803370	4.887199	-1.733127
C	-2.672437	-3.700893	0.342877
H	-1.879740	-4.460388	0.319829
H	-3.504716	-4.036309	0.969137
H	-3.019657	-3.501515	-0.680511
N	1.223955	2.308666	2.175987

47
C3 SCF Done: -1682.03329229 A.U.

C	-1.141034	1.019244	-0.195453
C	0.796364	0.496416	0.367639
C	0.154362	-0.905362	0.140435
C	-1.244878	-0.501927	-0.403283
H	0.098988	-1.378802	1.129284
H	-1.290542	-0.692843	-1.484836
N	0.250120	1.258182	-0.904908
C	-2.466972	-1.117384	0.243021
C	-3.641501	-1.187405	-0.521829
C	-2.503466	-1.567853	1.567863
C	-4.821110	-1.690256	0.022598
H	-3.622976	-0.840451	-1.557760
C	-3.684953	-2.074748	2.113180
H	-1.610467	-1.534020	2.194074
C	-4.846351	-2.136429	1.345101
H	-5.723054	-1.738396	-0.590364
H	-3.692754	-2.424555	3.147090
H	-5.767885	-2.534521	1.773621
C	2.211836	0.664249	0.801103
C	2.457431	1.071973	2.120756
C	3.325257	0.446006	-0.025711
C	3.751734	1.243478	2.602815
H	1.608441	1.253243	2.782237
C	4.625694	0.623759	0.442762
H	3.908013	1.555740	3.636120
H	5.463814	0.448159	-0.232316
C	0.894127	-1.877211	-0.750248
C	-2.226176	1.890734	-0.716173
O	-2.604759	2.825113	0.140569
O	1.910758	-2.434022	-0.102041
O	-2.664895	1.773470	-1.836038
O	0.593111	-2.155625	-1.884045
C	-3.614216	3.728794	-0.313657
H	-3.804855	4.413740	0.517660
H	-3.257981	4.281547	-1.192833
H	-4.526446	3.175031	-0.572240
C	2.692198	-3.378967	-0.830516
H	3.125662	-2.905622	-1.721615
H	3.484254	-3.707905	-0.150782
H	2.069365	-4.231440	-1.133010
N	0.639331	1.862298	-1.828117
C	-0.366352	1.204628	1.106594
H	-0.759752	0.682144	1.986547
H	-0.145217	2.257818	1.319020
C	4.839213	1.020630	1.760280
H	5.859306	1.156252	2.122748
Cl	3.137469	-0.057894	-1.685451

47

TS-CD3 SCF Done: -1682.02512129 A.U.

C	-1.341747	1.125076	-0.096769
C	0.818022	0.538392	0.352747
C	0.098929	-0.773681	0.036861
C	-1.333118	-0.356104	-0.412526
H	0.059690	-1.284226	1.013924
H	-1.449261	-0.509502	-1.496177
N	0.433570	1.345781	-1.345790
C	-2.447220	-1.123165	0.278304

C	-3.549131	-1.553099	-0.470722
C	-2.439814	-1.362439	1.658994
C	-4.614942	-2.213174	0.141361
H	-3.569019	-1.361978	-1.546264
C	-3.503501	-2.023502	2.272822
H	-1.598155	-1.026212	2.270189
C	-4.594996	-2.451851	1.515620
H	-5.464169	-2.544073	-0.459714
H	-3.479480	-2.203264	3.349357
H	-5.427430	-2.969185	1.996112
C	2.204157	0.624382	0.848662
C	2.400256	1.083966	2.166449
C	3.354668	0.292851	0.108752
C	3.668890	1.167113	2.727516
H	1.532911	1.356151	2.768169
C	4.629500	0.383517	0.661241
H	3.780142	1.511855	3.756239
H	5.493443	0.126107	0.047836
C	0.772473	-1.743694	-0.910139
C	-2.402809	1.978365	-0.524370
O	-2.338498	3.225408	-0.005505
O	1.675164	-2.487198	-0.285021
O	-3.281531	1.660630	-1.320005
O	0.501844	-1.856470	-2.078751
C	-3.338136	4.135333	-0.439552
H	-3.144685	5.077954	0.083670
H	-3.280043	4.292177	-1.526047
H	-4.341522	3.765134	-0.185941
C	2.362897	-3.454881	-1.077325
H	2.897760	-2.962112	-1.900017
H	3.070149	-3.950651	-0.405523
H	1.648749	-4.181830	-1.486475
N	0.813885	1.768614	-2.299985
C	-0.273417	1.426237	0.884224
H	-0.518264	1.035046	1.891590
H	0.010719	2.482158	0.969570
C	4.787896	0.811000	1.976440
H	5.788832	0.874587	2.405445
Cl	3.249910	-0.160689	-1.570753

50

C4 SCF Done: -1721.37233116 A.U.

C	1.187968	-1.164345	0.287453
C	-0.937722	-0.396554	0.818363
C	-0.096373	0.859184	0.432681
C	1.209225	0.295569	-0.179950
H	0.111934	1.379378	1.380377
H	1.083772	0.267698	-1.272457
N	-0.284378	-1.501545	-0.074514
C	2.472848	1.072112	0.117930
C	3.445487	1.184673	-0.885580
C	2.720684	1.673417	1.358843
C	4.636187	1.870907	-0.654342
H	3.259913	0.725190	-1.858593
C	3.913979	2.358796	1.592745
H	1.979656	1.619060	2.159203
C	4.875676	2.459368	0.588215
H	5.378323	1.950432	-1.450966

H	4.087635	2.820839	2.566349
H	5.806566	2.999305	0.770996
C	-2.430764	-0.391502	0.687058
C	-3.235684	-0.524970	1.825266
C	-3.090894	-0.267150	-0.548415
C	-4.627110	-0.538640	1.744411
H	-2.763829	-0.610768	2.804183
C	-4.478956	-0.289040	-0.646652
H	-5.219786	-0.637421	2.654741
H	-4.947430	-0.189722	-1.626289
C	-0.679651	1.952003	-0.436382
C	2.060285	-2.102662	-0.500368
O	2.173029	-3.287308	0.074024
O	-1.846096	2.386898	0.020663
O	2.538834	-1.829584	-1.574082
O	-0.113633	2.440196	-1.383497
C	2.886959	-4.282786	-0.660536
H	2.887143	-5.180107	-0.034836
H	2.379517	-4.479725	-1.614205
H	3.914539	-3.947363	-0.851831
C	-2.494278	3.397891	-0.748209
H	-2.670720	3.034879	-1.770518
H	-3.445487	3.599983	-0.245969
H	-1.878654	4.306482	-0.782897
N	-0.742712	-2.376257	-0.722442
C	1.115849	-1.293194	1.807954
H	1.314740	-2.326641	2.111794
H	1.855371	-0.643829	2.291561
C	-0.351491	-0.889086	2.151353
H	-0.402909	-0.110717	2.923053
H	-0.912533	-1.767717	2.494516
C	-5.250849	-0.427726	0.505263
H	-6.338945	-0.442090	0.426180
Cl	-2.200853	-0.014220	-2.031481

50

TS-CD4 SCF Done: -1721.34694665 A.U.

C	-1.466699	1.246065	0.479072
C	1.058558	0.237859	0.962922
C	0.043833	-0.767256	0.542594
C	-1.261743	-0.112787	-0.092945
H	-0.293574	-1.184272	1.510235
H	-1.106637	-0.025638	-1.179576
N	0.537524	1.856812	-0.449978
C	-2.474640	-1.010596	0.118805
C	-3.368852	-1.167045	-0.949614
C	-2.773624	-1.629399	1.338135
C	-4.527699	-1.926951	-0.805714
H	-3.147308	-0.681119	-1.902673
C	-3.935674	-2.390709	1.482981
H	-2.107484	-1.524472	2.198033
C	-4.816402	-2.543502	0.413357
H	-5.207648	-2.041387	-1.652259
H	-4.150324	-2.866621	2.441791
H	-5.722916	-3.140574	0.527688
C	2.488327	0.247635	0.661218
C	3.381261	0.578750	1.703014
C	3.064905	-0.037109	-0.598579

C	4.757998	0.595127	1.513114
H	2.978434	0.789052	2.693847
C	4.439831	-0.016407	-0.798169
H	5.414459	0.838864	2.349261
H	4.837873	-0.232645	-1.790073
C	0.460161	-1.996359	-0.238288
C	-2.027694	2.261335	-0.354370
O	-2.051060	3.488640	0.216486
O	1.581516	-2.528800	0.230109
O	-2.420377	2.096833	-1.507965
O	-0.187208	-2.488325	-1.128067
C	-2.550073	4.543338	-0.590839
H	-2.499840	5.450319	0.021468
H	-1.936948	4.668207	-1.494667
H	-3.590445	4.351971	-0.889822
C	2.065859	-3.686711	-0.448450
H	2.248398	-3.452800	-1.506623
H	3.001221	-3.961467	0.048697
H	1.337399	-4.504998	-0.374542
N	0.909448	2.664738	-1.103357
C	-1.039586	1.417937	1.880646
H	-1.229083	2.429713	2.255553
H	-1.571230	0.708532	2.531623
C	0.525099	1.089900	2.054521
H	0.585174	0.520059	2.994823
H	1.124431	2.003117	2.162881
C	5.291409	0.292214	0.261972
H	6.370231	0.300264	0.100241
Cl	2.067453	-0.352571	-1.992315

53
C5 SCF Done: -1760.67805426 A.U.

C	-1.283170	1.027629	0.414950
C	1.008527	0.453788	0.931827
C	0.185362	-0.832399	0.576381
C	-1.072848	-0.371332	-0.176435
H	-0.085365	-1.297604	1.533935
H	-0.811902	-0.234957	-1.234654
N	0.165711	1.572351	0.300804
C	-2.268529	-1.302119	-0.121415
C	-3.263746	-1.164412	-1.101786
C	-2.431189	-2.292705	0.854476
C	-4.393953	-1.978673	-1.095632
H	-3.141268	-0.409993	-1.881244
C	-3.562576	-3.112056	0.859660
H	-1.673871	-2.449002	1.624153
C	-4.549637	-2.956045	-0.110908
H	-5.154055	-1.853565	-1.869137
H	-3.666355	-3.879123	1.629344
H	-5.432636	-3.597684	-0.106372
C	2.430880	0.462613	0.405659
C	3.495866	0.286814	1.300335
C	2.761477	0.525438	-0.960200
C	4.816982	0.190759	0.872929
H	3.288088	0.198437	2.365987
C	4.083312	0.450302	-1.401101
H	5.611528	0.045072	1.605924
H	4.289470	0.516686	-2.469785

C	0.944035	-1.907027	-0.171618
C	-2.078725	1.970835	-0.462631
O	-2.559342	3.002368	0.206491
O	1.945280	-2.388367	0.556282
O	-2.182212	1.838929	-1.657480
O	0.675009	-2.307878	-1.276225
C	-3.189913	4.022503	-0.569121
H	-3.524134	4.782999	0.142572
H	-2.467750	4.449290	-1.277872
H	-4.045062	3.607211	-1.117903
C	2.809811	-3.322147	-0.087494
H	3.254089	-2.867092	-0.984291
H	3.589150	-3.568894	0.640098
H	2.253874	-4.225019	-0.373299
N	0.526305	2.632540	-0.098622
C	-1.638554	0.993976	1.919416
H	-2.504509	1.636578	2.115741
H	-1.936557	-0.029674	2.187757
C	-0.456599	1.453282	2.779407
H	-0.339137	2.537009	2.631238
H	-0.689784	1.302898	3.841838
C	0.884526	0.772885	2.443079
H	1.023175	-0.160420	3.007565
H	1.690036	1.458743	2.734281
C	5.115466	0.281535	-0.484141
H	6.146166	0.216035	-0.835725
Cl	1.551294	0.665103	-2.214811

53

TS-CD5 SCF Done: -1760.64348261 A.U.

C	-1.587631	1.208841	0.403214
C	1.187959	0.159370	1.050493
C	0.151057	-0.768976	0.575961
C	-1.186808	-0.123896	-0.084988
H	-0.227969	-1.223880	1.508622
H	-0.963189	-0.031115	-1.156585
N	0.589008	2.015950	-0.231277
C	-2.347684	-1.106625	0.048608
C	-3.137811	-1.327604	-1.090342
C	-2.713900	-1.733214	1.244335
C	-4.250027	-2.162988	-1.039161
H	-2.867425	-0.833462	-2.026757
C	-3.831540	-2.571558	1.296346
H	-2.139268	-1.577982	2.159552
C	-4.602093	-2.792896	0.157442
H	-4.843954	-2.327260	-1.940510
H	-4.096994	-3.052399	2.239898
H	-5.472692	-3.449846	0.199427
C	2.577715	0.217968	0.603633
C	3.588594	0.497832	1.553364
C	3.014712	0.048446	-0.734470
C	4.932820	0.545208	1.210468
H	3.316343	0.647263	2.596460
C	4.357370	0.111216	-1.087272
H	5.677068	0.738587	1.983756
H	4.637227	-0.014467	-2.133546
C	0.571222	-1.978598	-0.245755
C	-2.048685	2.126159	-0.590508

O	-2.496998	3.303544	-0.079985
O	1.712178	-2.500812	0.186928
O	-2.031339	1.954254	-1.811916
O	-0.083514	-2.471117	-1.128590
C	-2.929708	4.268915	-1.023235
H	-3.259086	5.139502	-0.445090
H	-2.109113	4.557139	-1.695858
H	-3.764321	3.884851	-1.627153
C	2.195617	-3.641835	-0.521000
H	2.350405	-3.389277	-1.579247
H	3.145585	-3.911443	-0.049491
H	1.479549	-4.470592	-0.443375
N	0.917773	2.852905	-0.867003
C	-1.682825	1.536695	1.868712
H	-1.596021	2.627519	1.973757
H	-2.684839	1.282490	2.270387
C	-0.635868	0.875977	2.775712
H	-0.662975	1.399499	3.742326
H	-0.923415	-0.157237	3.017214
C	0.828612	0.897107	2.318806
H	1.420675	0.418438	3.120182
H	1.208521	1.928550	2.256366
C	5.324381	0.346389	-0.112419
H	6.377307	0.385919	-0.394738
Cl	1.882096	-0.160666	-2.040400

56
C6 SCF Done: -1799.97585411 A.U.

C	1.163248	-1.039184	0.084408
C	-1.009816	-0.232466	0.907323
C	-0.118490	1.004561	0.564991
C	1.135806	0.474422	-0.176162
H	0.165966	1.453449	1.527051
H	0.936051	0.584539	-1.249734
N	-0.331289	-1.361885	0.080043
C	2.442579	1.186084	0.102395
C	3.387119	1.259539	-0.931749
C	2.769618	1.740581	1.345520
C	4.625299	1.866364	-0.729673
H	3.139570	0.835490	-1.908035
C	4.008893	2.350699	1.549180
H	2.059498	1.706371	2.174542
C	4.940809	2.414943	0.514278
H	5.344312	1.915830	-1.549581
H	4.243414	2.779614	2.525204
H	5.908001	2.894740	0.674844
C	-2.475600	-0.223303	0.494694
C	-3.510216	-0.436909	1.412675
C	-2.873835	-0.048103	-0.845776
C	-4.854269	-0.444679	1.036666
H	-3.284825	-0.607239	2.462590
C	-4.207342	-0.049860	-1.239965
H	-5.620242	-0.609756	1.795460
H	-4.451759	0.099757	-2.292026
C	-0.694906	2.196648	-0.172294
C	1.699922	-1.810666	-1.118022
O	2.176992	-2.997588	-0.791854
O	-1.889649	2.548767	0.281325

O	1.648840	-1.401564	-2.251529
O	-0.095894	2.822146	-1.012905
C	2.596233	-3.831569	-1.872860
H	2.956053	-4.759401	-1.418702
H	1.746330	-4.033134	-2.538442
H	3.400362	-3.342816	-2.438009
C	-2.514928	3.657916	-0.361884
H	-2.631581	3.452917	-1.435256
H	-3.494373	3.772249	0.112841
H	-1.914576	4.567301	-0.226328
N	-0.850304	-2.311449	-0.418716
C	-5.209005	-0.245173	-0.291593
H	-6.255526	-0.245655	-0.599636
Cl	-1.702780	0.205291	-2.121963
C	1.758946	-1.494122	1.434932
H	2.771830	-1.875378	1.249848
H	1.883892	-0.619233	2.083741
C	-0.758149	-0.616581	2.398859
H	-1.603853	-0.279535	3.006259
H	0.089442	-0.031959	2.772907
C	0.930124	-2.565704	2.153743
H	1.522813	-2.910298	3.012615
H	0.819335	-3.434221	1.488055
C	-0.470530	-2.095801	2.653379
H	-0.540241	-2.261251	3.737537
H	-1.262841	-2.708890	2.197644

56
TS-CD6 SCF Done: -1799.95441124 A.U.

C	-1.595001	1.258040	0.146737
C	1.163925	-0.074758	1.091206
C	0.055206	-0.848744	0.552176
C	-1.262463	-0.105943	-0.227447
H	-0.443226	-1.293104	1.424501
H	-0.945179	-0.103945	-1.273988
N	0.703981	1.976991	0.287588
C	-2.443267	-1.063483	-0.112308
C	-3.211366	-1.245997	-1.273081
C	-2.841112	-1.721565	1.055126
C	-4.335511	-2.066200	-1.270100
H	-2.913029	-0.730496	-2.189890
C	-3.972247	-2.544633	1.061365
H	-2.282910	-1.603459	1.985778
C	-4.721254	-2.723084	-0.098384
H	-4.912340	-2.196510	-2.187835
H	-4.263426	-3.047478	1.985555
H	-5.601186	-3.368876	-0.092489
C	2.524670	-0.097037	0.525737
C	3.628822	-0.192023	1.400528
C	2.829896	-0.039234	-0.853064
C	4.935821	-0.272334	0.936167
H	3.456737	-0.239507	2.474679
C	4.137154	-0.108623	-1.327021
H	5.754206	-0.366525	1.651162
H	4.314839	-0.049793	-2.401271
C	0.433115	-2.040680	-0.322255
C	-1.526327	2.246219	-0.909836
O	-1.756440	3.503934	-0.471348

O	1.449266	-2.720394	0.200024
O	-1.256789	2.036900	-2.087069
O	-0.136393	-2.392164	-1.323648
C	-1.692594	4.532389	-1.447532
H	-1.906152	5.469377	-0.921843
H	-0.692654	4.576807	-1.901914
H	-2.437362	4.368877	-2.239289
C	1.896747	-3.856883	-0.537311
H	2.218659	-3.549572	-1.541985
H	2.740520	-4.269690	0.024094
H	1.092078	-4.599295	-0.621744
N	1.233792	2.916093	0.060330
C	1.037480	0.413004	2.511768
H	1.493622	-0.383855	3.130265
H	1.681799	1.295943	2.648642
C	5.195981	-0.237430	-0.432909
H	6.218079	-0.298587	-0.809125
Cl	1.582048	0.209707	-2.042977
C	-2.157349	1.604098	1.489418
H	-2.801219	0.774495	1.829119
H	-2.803073	2.487432	1.393051
C	-0.340783	0.681486	3.124363
H	-0.139761	0.831379	4.195809
H	-0.968705	-0.222239	3.081459
C	-1.152511	1.887426	2.629859
H	-1.750334	2.251633	3.479927
H	-0.488565	2.720099	2.349600

59

C7 SCF Done: -1839.27837256 A.U.

C	-1.204890	-0.926907	0.061417
C	1.039198	-0.304901	-0.781553
C	0.148574	0.977046	-0.660727
C	-1.099387	0.604310	0.176835
H	-0.144807	1.242976	-1.687541
H	-0.859109	0.812810	1.226698
N	0.287447	-1.294208	0.137985
C	-2.375991	1.356371	-0.139580
C	-3.311909	1.528188	0.891104
C	-2.683292	1.859319	-1.409153
C	-4.522987	2.177159	0.658855
H	-3.079451	1.147602	1.888494
C	-3.894750	2.513034	-1.642763
H	-1.979834	1.748933	-2.236451
C	-4.819470	2.672442	-0.611908
H	-5.235727	2.301596	1.476281
H	-4.113165	2.900299	-2.639720
H	-5.765383	3.185037	-0.796110
C	2.480676	-0.223420	-0.286767
C	3.558315	-0.497888	-1.138673
C	2.819176	0.125185	1.034341
C	4.885539	-0.416507	-0.718212
H	3.375696	-0.785508	-2.171638
C	4.137534	0.212995	1.472640
H	5.685570	-0.636880	-1.426094
H	4.335693	0.494548	2.507206
C	0.768478	2.261851	-0.150716
C	-1.784034	-1.571573	1.313246

O	-2.248901	-2.785267	1.074792
O	1.939743	2.516739	-0.718065
O	-1.782495	-1.057842	2.404087
O	0.230502	3.015025	0.623458
C	-2.716017	-3.520478	2.206654
H	-3.055694	-4.486758	1.822437
H	-1.896403	-3.658638	2.924451
H	-3.544342	-2.986285	2.689801
C	2.617781	3.693244	-0.280622
H	2.797216	3.641768	0.802354
H	3.567649	3.716550	-0.823634
H	2.022271	4.586495	-0.511299
N	0.770103	-2.139876	0.826141
C	5.181094	-0.056947	0.591481
H	6.214040	0.013509	0.935314
Cl	1.598559	0.506861	2.228271
C	1.047080	-2.389582	-2.394497
C	-1.895460	-1.386095	-1.235956
C	-0.097005	-3.275231	-1.852301
C	-1.535473	-2.735411	-1.899742
H	-1.782165	-0.579054	-1.966933
H	1.997888	-2.746329	-1.965557
H	0.119062	-3.602103	-0.828100
H	1.124387	-2.552595	-3.480751
H	-2.973549	-1.397562	-1.012327
H	-0.096139	-4.201291	-2.447749
H	-2.174126	-3.517647	-1.464317
H	-1.849399	-2.643427	-2.952396
C	0.921197	-0.871534	-2.216985
H	-0.032306	-0.555956	-2.649681
H	1.669126	-0.357390	-2.834910

59

TS-CD7 SCF Done: -1839.24685543 A.U.

C	-1.678669	-0.969962	-0.040640
C	1.214217	-0.203783	-0.892406
C	0.189536	0.834832	-0.656434
C	-1.163019	0.398573	0.158647
H	-0.193445	1.053851	-1.666925
H	-0.890811	0.501102	1.218000
N	0.560526	-1.613331	0.670921
C	-2.265663	1.414328	-0.136645
C	-3.094357	1.779743	0.934991
C	-2.538221	1.946208	-1.400284
C	-4.157985	2.658367	0.751412
H	-2.893832	1.361346	1.924977
C	-3.607976	2.827120	-1.586614
H	-1.921929	1.687977	-2.264496
C	-4.419952	3.189037	-0.514654
H	-4.785010	2.933282	1.601906
H	-3.801505	3.232011	-2.581912
H	-5.252262	3.879570	-0.661915
C	2.602008	-0.193915	-0.409538
C	3.619014	-0.724579	-1.236401
C	3.025916	0.271333	0.860254
C	4.953799	-0.749518	-0.853446
H	3.363671	-1.105718	-2.223431
C	4.359179	0.244652	1.253891

H	5.699931	-1.153794	-1.538442
H	4.625866	0.613788	2.244478
C	0.643746	2.197573	-0.150242
C	-2.042314	-1.699181	1.142256
O	-2.686115	-2.861362	0.862563
O	1.800244	2.560602	-0.694459
O	-1.811205	-1.386816	2.309103
O	0.013439	2.915082	0.583594
C	-3.067888	-3.651342	1.977389
H	-3.564306	-4.539244	1.570691
H	-2.188574	-3.950342	2.565134
H	-3.761221	-3.101198	2.629434
C	2.327089	3.820231	-0.281158
H	2.485471	3.822427	0.806366
H	3.280467	3.937674	-0.805391
H	1.637949	4.630966	-0.552054
N	0.937232	-2.132419	1.567685
C	5.332154	-0.258451	0.394077
H	6.376779	-0.271881	0.707673
Cl	1.893045	0.859072	2.048463
C	0.913951	-2.608900	-1.992569
C	-2.206729	-1.409858	-1.380963
C	-0.363090	-3.319790	-1.508923
C	-1.713289	-2.765839	-1.972665
H	-2.010604	-0.602612	-2.098817
H	1.774328	-2.932510	-1.384192
H	-0.388254	-3.407914	-0.415713
H	1.118498	-2.957496	-3.016363
H	-3.308551	-1.475792	-1.323451
H	-0.297110	-4.355588	-1.879098
H	-2.462803	-3.531261	-1.725551
H	-1.721967	-2.676446	-3.072258
C	0.873056	-1.072722	-2.077431
H	-0.102386	-0.768103	-2.466257
H	1.577567	-0.753082	-2.864330

62
C8 SCF Done: -1878.58678264 A.U.

C	1.244402	0.761866	0.190464
C	-1.051684	0.320638	-0.682851
C	-0.197648	-0.983915	-0.732501
C	1.048863	-0.767181	0.143454
H	0.084741	-1.132580	-1.783986
H	0.784068	-1.071982	1.161479
N	-0.218582	1.242642	0.228337
C	2.279159	-1.561958	-0.242430
C	3.154879	-1.959584	0.778122
C	2.602160	-1.890634	-1.564793
C	4.324091	-2.660374	0.487059
H	2.909523	-1.713192	1.813981
C	3.771291	-2.594969	-1.857658
H	1.946708	-1.596967	-2.387025
C	4.636545	-2.981063	-0.834629
H	4.990459	-2.962049	1.297357
H	4.004580	-2.842737	-2.894879
H	5.549289	-3.533159	-1.066096
C	-2.451894	0.177868	-0.086490
C	-3.585052	0.367254	-0.891097

C	-2.700249	-0.193253	1.249191
C	-4.881233	0.184665	-0.415392
H	-3.465610	0.663005	-1.931611
C	-3.992792	-0.377082	1.741249
H	-5.726012	0.340667	-1.087521
H	-4.124388	-0.664665	2.784740
C	-0.941220	-2.249511	-0.364487
C	1.810430	1.200578	1.539256
O	2.377149	2.392115	1.493779
O	-1.953604	-2.467307	-1.197487
O	1.722465	0.547607	2.549477
O	-0.659082	-2.990708	0.543306
C	2.849278	2.918221	2.734677
H	3.285537	3.894656	2.504043
H	2.011329	3.027642	3.436001
H	3.606499	2.250897	3.166237
C	-2.806860	-3.567728	-0.889638
H	-3.250130	-3.432001	0.107189
H	-3.588292	-3.572024	-1.655817
H	-2.242146	-4.509113	-0.913913
N	-0.643614	2.137979	0.896044
C	-5.090216	-0.193562	0.907199
H	-6.098751	-0.341938	1.295837
Cl	-1.414528	-0.449601	2.404185
C	-1.444098	2.444272	-2.154773
C	2.014376	1.301542	-1.025757
C	0.783366	3.676677	-1.434493
C	2.046118	2.801055	-1.375110
H	1.688162	0.723145	-1.899888
H	-1.902329	2.767402	-1.205374
H	0.316439	3.751781	-0.441396
H	-2.220105	2.571689	-2.924463
H	3.059719	0.988379	-0.871271
H	1.167811	4.687480	-1.644055
H	2.738125	3.296142	-0.681211
H	2.530952	2.854362	-2.364566
C	-1.062031	0.959847	-2.086222
H	-0.068790	0.828682	-2.528489
H	-1.716809	0.334092	-2.709123
C	-0.285402	3.388835	-2.504696
H	-0.739951	4.355411	-2.772371
H	0.208821	3.027159	-3.424160

62
TS-CD8 SCF Done: -1878.55157217 A.U.

C	-1.735174	-0.756557	0.114565
C	1.248934	-0.260561	-0.763607
C	0.274852	0.847801	-0.661094
C	-1.109995	0.584226	0.163322
H	-0.072855	0.982808	-1.700035
H	-0.835049	0.777643	1.208003
N	0.456866	-1.518236	0.831496
C	-2.131819	1.641915	-0.248057
C	-2.926739	2.192558	0.768459
C	-2.366872	2.040095	-1.567166
C	-3.920835	3.121970	0.476589
H	-2.754154	1.880454	1.802016
C	-3.365788	2.973068	-1.862052

H	-1.777672	1.630177	-2.390880
C	-4.144443	3.519097	-0.844587
H	-4.522827	3.541943	1.284879
H	-3.532002	3.271199	-2.899098
H	-4.922289	4.248734	-1.076660
C	2.635234	-0.246440	-0.287327
C	3.630281	-0.784115	-1.131357
C	3.074992	0.270191	0.952125
C	4.973374	-0.788475	-0.777416
H	3.340930	-1.174229	-2.106288
C	4.415628	0.256262	1.321494
H	5.711119	-1.197240	-1.468821
H	4.703695	0.658797	2.292918
C	0.813277	2.217789	-0.267087
C	-2.125623	-1.293999	1.395275
O	-2.940147	-2.371728	1.297962
O	1.972821	2.475419	-0.862290
O	-1.780411	-0.886956	2.502356
O	0.249299	3.015850	0.436842
C	-3.351168	-2.960602	2.521734
H	-3.989770	-3.809978	2.255270
H	-2.483766	-3.310545	3.098712
H	-3.918147	-2.242936	3.131443
C	2.582071	3.727598	-0.551309
H	2.771290	3.795999	0.529012
H	3.525082	3.752335	-1.105922
H	1.932406	4.556476	-0.861998
N	0.783510	-2.021447	1.757470
C	5.370359	-0.269907	0.454148
H	6.421101	-0.273259	0.747203
Cl	1.950428	0.930993	2.107764
C	1.220227	-2.711083	-1.768104
C	-2.422942	-1.234306	-1.146617
C	-1.169084	-3.611549	-1.191716
C	-2.401907	-2.742973	-1.501063
H	-2.028681	-0.648746	-1.989636
H	1.565259	-2.986244	-0.759277
H	-0.827973	-3.418131	-0.161916
H	2.059994	-2.926893	-2.444184
H	-3.491391	-0.946311	-1.096580
H	-1.543648	-4.646680	-1.178027
H	-3.246592	-3.208894	-0.977245
H	-2.634226	-2.830410	-2.575944
C	0.845279	-1.227661	-1.835107
H	-0.243547	-1.154159	-1.892951
H	1.189620	-0.776770	-2.786451
C	0.031226	-3.593244	-2.163279
H	0.406496	-4.622888	-2.265976
H	-0.314871	-3.298625	-3.169889

44

TS-DD’ SCF Done: -1534.44646071 A.U.

C	0.686234	-1.694406	0.376282
C	-0.913037	0.701904	-1.090027
C	-0.366997	-0.570717	-1.660403
C	0.940510	-1.070864	-0.953081
H	-0.224288	-2.269349	0.535560
H	-0.604860	1.613367	-1.597845

H	-0.058107	-0.337627	-2.693263
H	1.318381	-1.878761	-1.608121
C	1.959295	0.055666	-0.957437
C	2.908395	0.137204	-1.978390
C	1.908772	1.073798	0.004243
C	3.796533	1.213939	-2.039865
H	2.953618	-0.651787	-2.733537
C	2.790157	2.150319	-0.056924
H	1.173380	1.019021	0.812233
C	3.737587	2.224119	-1.081344
H	4.535838	1.261240	-2.841739
H	2.735408	2.937746	0.697390
H	4.428973	3.067518	-1.128962
C	-1.618444	0.849749	0.136911
C	-1.758796	2.128480	0.753032
C	-2.213613	-0.236455	0.835233
C	-2.430994	2.308312	1.952605
C	-2.886818	-0.060928	2.035975
H	-2.175029	-1.240240	0.412822
H	-2.500648	3.308467	2.381829
H	-3.333043	-0.924219	2.531695
C	-1.363014	-1.700334	-1.851291
C	1.695089	-1.834913	1.415062
O	1.179720	-2.387919	2.524332
O	-2.571083	-1.255778	-2.170757
O	2.867512	-1.522607	1.331728
O	-1.090185	-2.874257	-1.772582
C	2.084020	-2.581802	3.605021
H	1.502116	-3.034201	4.414349
H	2.903348	-3.250942	3.307697
H	2.504823	-1.621006	3.932658
C	-3.572676	-2.245558	-2.405916
H	-3.719993	-2.855923	-1.504540
H	-4.488602	-1.698626	-2.649019
H	-3.279514	-2.892591	-3.243151
C	-3.000945	1.210774	2.601820
H	-3.529331	1.355163	3.545051
Cl	-1.030454	3.533384	0.016358

44
TS-DD'' SCF Done: -1534.43838311 A.U.

C	1.937196	-1.582134	0.627153
C	-0.697644	1.113779	1.033222
C	0.570421	0.529512	1.593272
C	0.784824	-1.031808	1.405684
H	2.144482	-2.636961	0.817805
H	-1.386221	1.517271	1.773869
H	0.453367	0.632899	2.682405
H	0.982562	-1.402305	2.424819
C	-0.527900	-1.694972	0.989185
C	-1.606248	-1.677170	1.883253
C	-0.720867	-2.223120	-0.289896
C	-2.859866	-2.143968	1.494758
H	-1.464077	-1.270034	2.888186
C	-1.975692	-2.696934	-0.680534
H	0.105572	-2.243138	-1.004235
C	-3.050308	-2.648669	0.205918
H	-3.693638	-2.110064	2.198670

H	-2.112453	-3.095704	-1.687654
H	-4.033602	-3.008198	-0.102912
C	-1.162393	1.060474	-0.312531
C	-2.531639	1.265013	-0.636341
C	-0.304422	0.753180	-1.397720
C	-3.010515	1.154720	-1.935317
C	-0.775256	0.650895	-2.698470
H	0.754690	0.603124	-1.190842
H	-4.073151	1.306849	-2.128461
H	-0.079888	0.415000	-3.505903
C	1.849794	1.317640	1.375935
C	2.807757	-0.981346	-0.365889
O	3.749177	-1.847822	-0.764422
O	1.691225	2.403255	0.633722
O	2.741336	0.147420	-0.823223
O	2.897522	1.010123	1.894291
C	4.664051	-1.375924	-1.746715
H	5.342500	-2.208748	-1.956735
H	4.129892	-1.081474	-2.660613
H	5.228728	-0.514441	-1.364147
C	2.867162	3.170924	0.384263
H	3.607985	2.555133	-0.143364
H	2.554534	4.012397	-0.241660
H	3.294354	3.534649	1.328154
C	-2.131901	0.844432	-2.974914
H	-2.510921	0.758119	-3.994242
Cl	-3.684595	1.629686	0.624590

44
TS-DD''' SCF Done: -1534.43943354 A.U.

C	1.092014	0.317560	-1.630828
C	0.200009	-0.504818	1.453578
C	0.716323	-1.353166	0.327089
C	0.417466	-0.911043	-1.124905
H	0.749767	0.717172	-2.586344
H	0.152132	-1.007714	2.422870
H	0.200873	-2.329989	0.427984
H	0.840216	-1.729556	-1.741087
C	-1.082021	-0.916503	-1.405827
C	-1.751466	-2.146240	-1.465313
C	-1.819848	0.256728	-1.588789
C	-3.125466	-2.202835	-1.685133
H	-1.184547	-3.073011	-1.339209
C	-3.197411	0.203072	-1.809642
H	-1.324036	1.229455	-1.535171
C	-3.855399	-1.024645	-1.853962
H	-3.628429	-3.170830	-1.728128
H	-3.757595	1.131289	-1.940679
H	-4.932893	-1.065172	-2.024480
C	-0.573113	0.741341	1.320055
C	-1.963444	0.747613	1.530854
C	0.026933	1.973433	1.011466
C	-2.729636	1.903867	1.423691
C	-0.726492	3.140599	0.890786
H	1.110803	2.011199	0.900974
H	-3.807725	1.853596	1.581286
H	-0.229527	4.081537	0.649298
C	2.175584	-1.766677	0.467073

C	2.293495	0.929010	-1.095287
O	2.756719	1.894307	-1.898809
O	2.641877	-1.625001	1.699425
O	2.835060	0.656391	-0.035752
O	2.821829	-2.249165	-0.433003
C	3.916059	2.587012	-1.448628
H	4.158203	3.315070	-2.229117
H	3.711906	3.102724	-0.499820
H	4.751885	1.888193	-1.308094
C	4.012628	-1.964561	1.895092
H	4.647325	-1.331071	1.260075
H	4.225630	-1.777614	2.952142
H	4.187339	-3.020958	1.651714
C	-2.105632	3.106311	1.093133
H	-2.701882	4.015648	1.001651
Cl	-2.772755	-0.761681	1.858956
